# Symmetry and Vibrational Control of Multi-Channel HHG in Methane

**DOI:** 10.1021/acs.jpclett.6c02522

**Published:** 2026-10-08

**Authors:** Marco Marchetta, Eleonora Luppi, Emanuele Coccia

**Affiliations:** † Dipartimento di Scienze Chimiche e Farmaceutiche, 9315 Università di Trieste, via L. Giorgieri 1, 34127 Trieste, Italy; ‡ Laboratoire de Chimie Théorique, 27063 Sorbonne Université, CNRS, Paris F-75005, France

## Abstract

High-harmonic generation (HHG) driven by tailored bicircular laser fields has emerged as a powerful tool for probing structural and dynamical properties of complex molecules. We compute HHG spectra of methane (CH_4_) interacting with intense ω/2ω counter-rotating bicircular fields using an all-electron approach based on the time-dependent Schrödinger equation. The emitted harmonics are explained as the interplay between the dynamical symmetries of the driving field and the tetrahedral spatial symmetry of the molecule. We unravel the critical role of molecular normal modes in modulating population dynamics across distinct ionization and recombination channels. The appearance of forbidden harmonics under asymmetric stretching of methane is analyzed, showing a clear interplay between such harmonics and the spatial displacement. This work provides insights for experimental investigations of aligned molecular systems, with a view toward a pump–probe setup.

## Graphical abstract

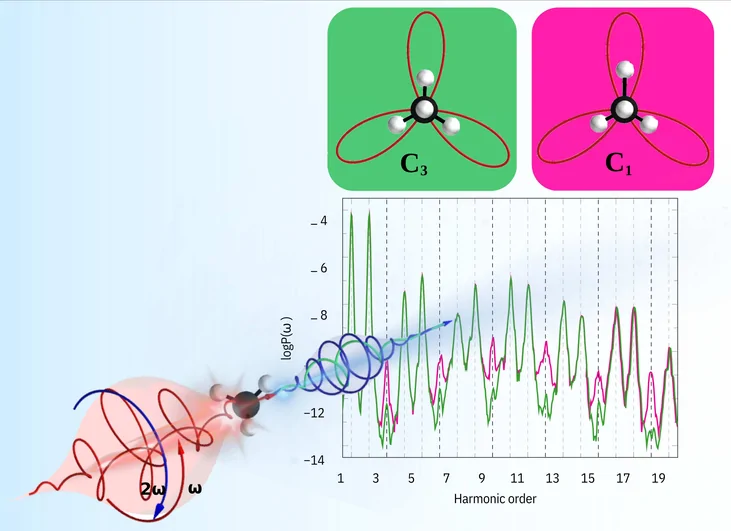

High-harmonic generation (HHG) is a nonlinear optical process widely employed to produce coherent ultrashort pulses in the extreme ultraviolet (XUV) region, used to investigate ultrafast phenomena through time-resolved spectroscopies.^1–3^ In addition, the HHG signal itself encodes valuable information about the atomic/molecular emitter. This phenomenon has been used for molecular imaging,^4–7^ to investigate electron dynamics by highlighting its multiorbital nature,^8–12^ to study vibrational dynamics,^13–17^ nonadiabatic effects,^18–20^ and to probe chirality in gas-phase media^21–26^ and solids.^27,28^

In this context, driving pulses with nontrivial polarization states are increasingly used to tailor the properties of the emitted harmonic radiation and control electron dynamics. One of the most widely investigated schemes is the bicircular counter-rotating (BCR) pulse, which enables the generation of circularly polarized harmonics^29–35^ and chiral HHG spectroscopy.^22,23,25–27^ This pulse consists of two circularly polarized counter-rotating pulses, one with frequency ω and the other with frequency *qω*, with *q* an integer. The resulting electric field, polarized in the **xy** plane, is

1 $$E\left(t\right)=E_{0}\left[\cos\left(\omega t\right)\mathrm{x̂}+\sin\left(\omega t\right)ŷ\right]+E_{0}^{\prime }\left[\cos\left(q\omega t\right)\mathrm{x̂}-\sin\left(q\omega t\right)ŷ\right]$$

While in principle any multiple *qω* could be employed, experiments are typically limited to the second harmonic, obtaining ω/2ω BCR pulses. The space-time symmetry of the pulse, or dynamical symmetry,^35^ enforces characteristic selection rules in isotropic media and in oriented systems that possess the same spatial symmetry.^35–38^ The ω/2ω BCR pulse defined in eq 1 possesses a C_3_ spatiotemporal symmetry, corresponding to invariance under a rotation by 2π/3 (*Ĉ*_3_) combined with a time translation of *T*/3 (τ̂_3_), where *T* is the fundamental period. For this case, harmonics with order 3N ± 1 are emitted in the field polarization plane with helicities ±1.^29–33,35,38–45^ Linearly polarized 3N harmonics can in principle be emitted perpendicular to the polarization plane,^46^ but in practice they are often suppressed due to unfavorable phase-matching conditions. The computed HHG spectra corresponding to these cases, together with the corresponding helicities, are reported in Figure S1 of the Supporting Information (SI).

In this work, we employ ab initio real-time time-dependent configuration interaction singles (RT-TD-CIS),^47–56^ combined with a Gaussian basis set optimized for the continuum,^57–60^ to study HHG in CH_4_ with ω/2ω BCR pulses in symmetric and distorted structures, the latter representative of the turning points of the relevant vibrational modes: CH_4_ in its equilibrium, symmetrically and asymmetrically stretched configurations corresponding to the A_1_ (3054.37 cm^–1^, Raman-active) and one of the T_2_ (3165.12 cm^–1^, IR-active) normal modes. RT-TD-CIS has proven to be a valid and computationally inexpensive approach for studying strong-field dynamics of polyatomic molecules,^54,61^ with RT-TD-CISD limited to few-electron systems.^62^

This investigation supports the idea of a pump–probe experiment, in which an IR pump vibrationally excites the molecule, while a subsequent intense driving pulse with a central wavelength of 800 nm triggers HHG^14–17^ to probe the resulting dynamical evolution. CH_4_ is used as a model molecule to study the interplay between normal modes and HHG spectra. The probe pulse has a peak intensity of 6.0 × 10^13^ W/cm^2^, 30 optical cycles and a central wavelength of 800 nm. The probe at 800 nm (12500 cm^–1^) does not affect the selected normal modes, whose energies are much lower.

CH_4_ belongs to the *T*_*d*_ group symmetry, which contains 4 C_3_ axes, coinciding with the 4 CH bonds. In Figure 1, we report the three systems investigated: CH_4_ at equilibrium geometry (Figure 1(a)), symmetrically stretched (Figure 1(b)), and asymmetrically stretched (Figure 1(c)). For the symmetrically stretched configuration, all CH bonds are displaced by 4 × 10^–3^ Å compared to equilibrium (green arrow). In the asymmetric configuration, one CH bond is displaced by 4 × 10^–3^ Å (green arrow) and the other three by 4/3 × 10^–3^ Å in the opposite direction (yellow arrow). The fixed-nuclei approximation is justified by the fact that in an ω/2ω BCR field, the harmonic emission occurs three times per fundamental laser cycle^29^ (∼0.9 fs).

**Figure 1:**
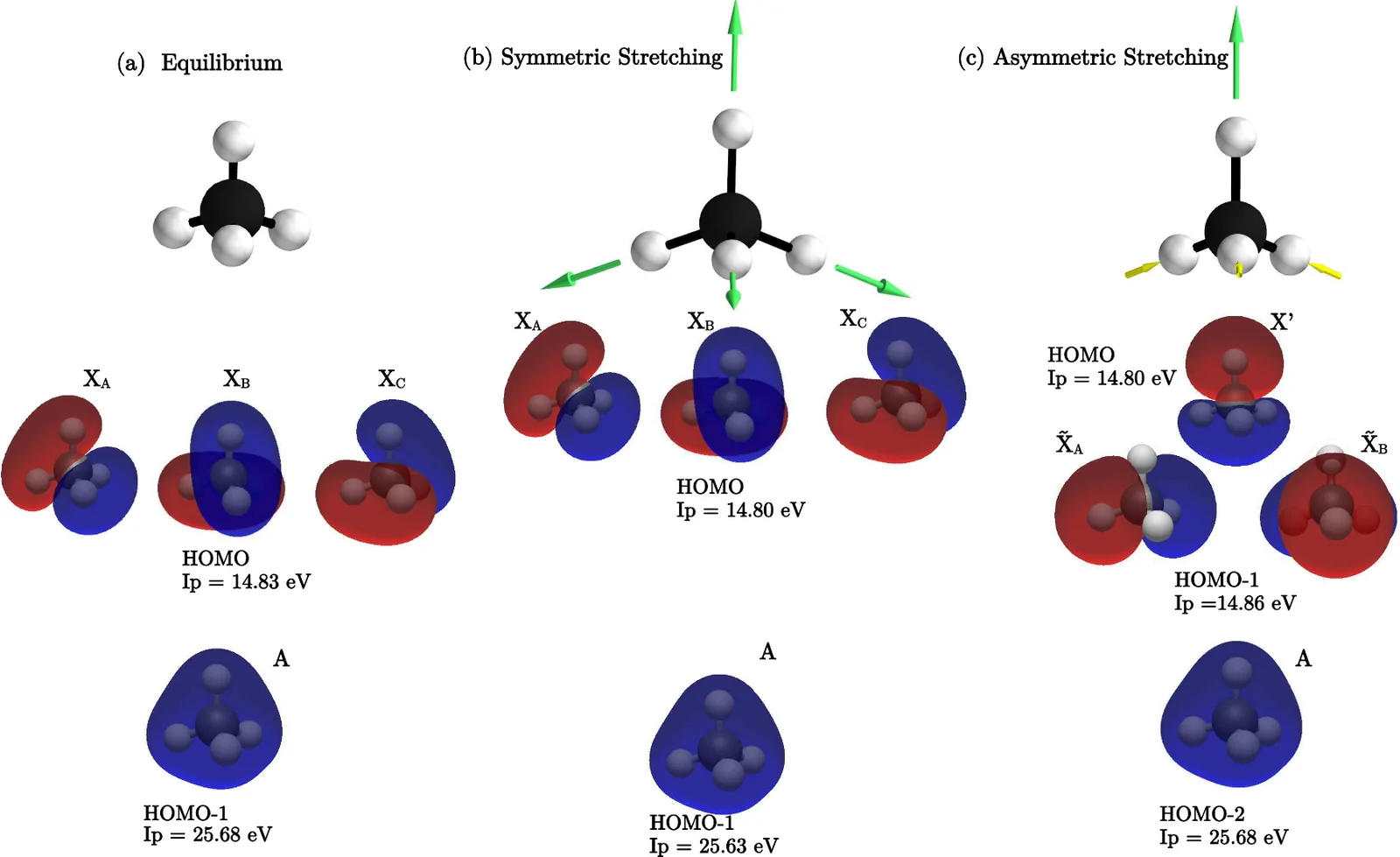
Representation of the CH_4_ molecule and the HF occupied MOs investigated in this study, for (a) the equilibrium geometry, (b) a symmetrically stretched configuration, and (c) an asymmetrically stretched configuration; the carbon atom is kept fixed at its equilibrium position, and the reported displacements refer purely to changes in the C–H bond lengths, not to a mass-weighted normal-mode eigenvector.

Moreover, since ionization and recombination can occur to different molecular orbitals (MOs), HHG spectra encode multichannel electron dynamics.^8,15,55,63–65^ We apply the MO decomposition of the HHG signal presented in refs 55. and^56^ to study the role of ionization-recombination channels. Ionization channels are typically described in terms of Dyson orbitals, defined as the overlap between the M-electron neutral wave function and the (M-1)-electron wave function of the corresponding cation. Although HF orbitals neglect both electronic correlation and orbital relaxation, they provide a first approximation to the Dyson orbitals and a useful description of the ionization channels,^55,66^ and have been used in this work. Details on the theory and simulations, including the impact of the ionization model for various parameters (Figure S2), are provided in the SI.

As expected, Figure 1 shows that symmetric stretching maintains the system symmetry and preserves the triple degeneracy of the HOMO, while asymmetric stretching reduces the symmetry to C_3*v*_. Consequently, the three degenerate orbitals split into a nondegenerate HOMO and a doubly degenerate HOMO–1. In the upper panel of Figure 2, we illustrate the emitter-pulse configurations together with the corresponding calculated HHG spectra, for three geometries of the molecule: equilibrium, symmetrically stretched, and asymmetrically stretched. In each configuration, the driving-field rotation axis is aligned with a C–H bond. At equilibrium and under symmetric stretching, all four C–H bonds are equivalent; under asymmetric stretching, the field rotational axis is aligned with one of the three compressed bonds. In the equilibrium and symmetrically stretched configurations, the molecular projection onto the pulse polarization plane is symmetric with respect to a C_3_ rotation, resulting in the emission of 3N ± 1 circularly polarized harmonics. In the asymmetrically stretched configuration, the C_3_ symmetry is broken and harmonics of all orders become allowed; however, due to the small atomic displacement, 3N harmonics are about 2–3 orders of magnitude weaker than the 3N ± 1 ones. Emission of 3N harmonics is observed only when the single emitter maintains a fixed orientation relative to the driving pulse. These harmonics are completely suppressed when averaging over the molecular orientation, as shown in the bottom panel of Figure 2, where we present the rotationally averaged spectrum of the asymmetrically stretched configuration corresponding to an elongation of 6 × 10^–3^ Å. Moreover, with a rotational constant of 5.32 cm^–1^ estimated from a rigid-rotor calculation, CH_4_ populates a very large number of rotational states at room temperature. The resulting near-isotropic orientational distribution is therefore equivalent, for HHG purposes, to the orientation averaging shown in the bottom panel of Figure 2.

**Figure 2:**
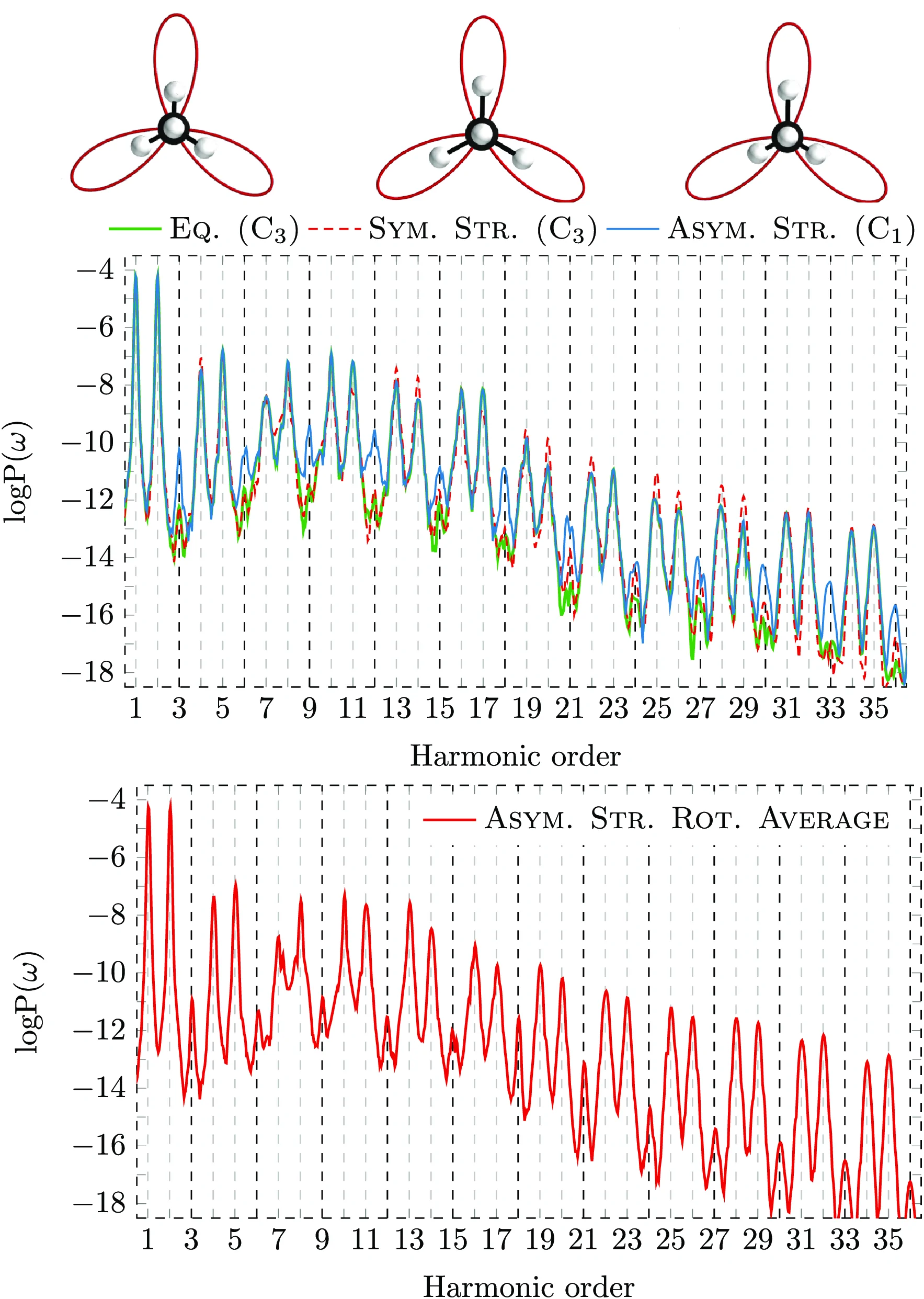
Top panel: comparison of HHG spectra of the equilibrium geometry, a symmetrically stretched configuration (all CH bonds elongated by 4 × 10^–3^ Å), and the asymmetrically stretched configuration (one CH bond elongated by 4 × 10^–3^ Å and the other three shortened by 4/3 × 10^–3^ Å). Bottom panel: the orientation-averaged spectrum of the asymmetrically stretched configuration (red) corresponding to an elongation of 6 × 10^–3^ Å and compression of 2 × 10^–3^ Å, with respect to equilibrium. For the rotationally averaged case we use a larger displacement for illustration purposes.

Having an all-electron first-principles approach, we combine selection-rule analysis with the role MOs play during the dynamics. In Figure 3, we report the MO decomposition of the HHG spectrum for the equilibrium configuration. While for the total spectrum and for the A channel only 3N ± 1 harmonics are emitted (bottom panel), for the contributions associated with each individual degenerate orbital, all harmonic orders are produced, since an individual member of the degenerate manifold does not possess C_3_ rotational symmetry (upper panel of Figure 3). Nevertheless, the partial contribution of one degenerate HOMO is not individually observable, and the representation of the three degenerate orbitals is arbitrary; information on ionization-recombination processes related to the physical X channel can be found only by considering all three contributions from the three degenerate MOs. Coherently summing the time-dependent dipoles corresponding to the three degenerate components of the HOMO, we obtain the net X channel signal, whose contribution to the spectra correctly recovers the 3N ± 1 selection rule (middle panel of Figure 3). Analyzing the channel contributions, we note that until the semiclassical cutoff (H17, qualitatively using the standard one-color equation^67^), the X spectrum overlaps with the total spectrum, while the A channel contributes negligibly. The ionization energy of a given channel is given by minus the energy of the corresponding orbital, according to Koopmans’ theorem. Although Koopmans’ theorem is not the most accurate method for evaluating ionization energies, the relative energy differences obtained here are in good agreement with literature values.^68^ The pronounced single-channel behavior can be readily explained by the large difference (10.85 eV) in the ionization energy of the two physical channels.

**Figure 3:**
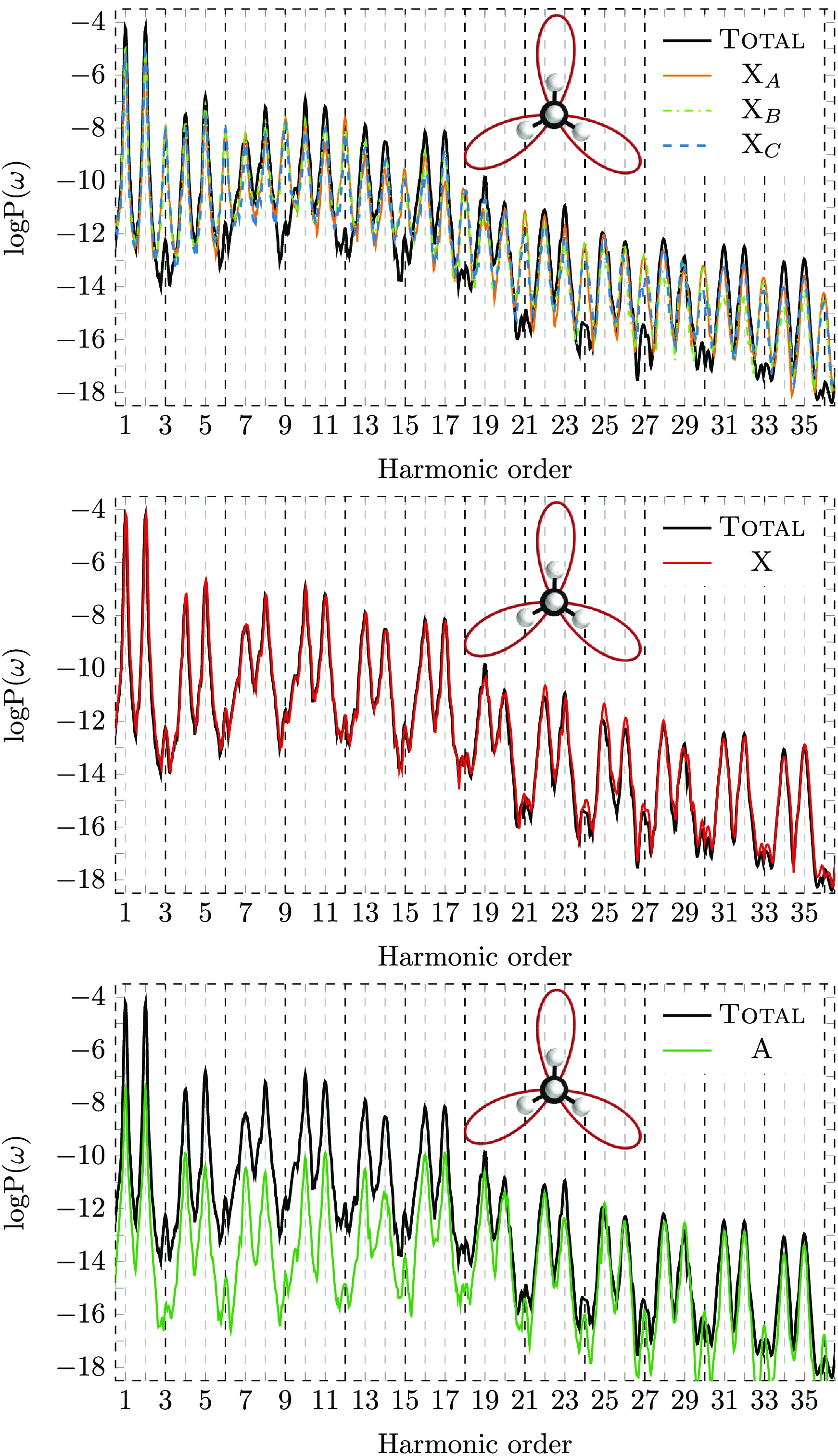
MO decomposition of HHG spectra of CH_4_ at its equilibrium configuration with pulse polarized perpendicularly to the CH bond. The top panel shows the individual contributions of the three MOs associated with the X channel. The middle panel displays their combined partial contributions, describing the overall physical X channel. The bottom panel displays the contribution of the A channel.

In contrast to the equilibrium configuration, where all CH bonds are equivalent, the asymmetrically stretched configuration yields two distinct pulse-molecule configurations when the pulse rotational axis aligns with the CH bond. In particular, the molecular projection onto the pulse polarization plane exhibits either C_3_ or C_1_ rotational symmetry. In Figure 4, we report the channel decomposition for the asymmetrically stretched configuration with C_1_ symmetry projection. Three physical channels are identified: X′ coincides with the nondegenerate HOMO channel, while X̃ is defined by the sum of the two degenerate X̃_A_ and X̃_B_ MOs describing HOMO–1, and A corresponds to HOMO–2 (Figure 1). In analogy with the equilibrium geometry, X̃_A_ and X̃_B_ alone do not represent individual ionization channels; rather, the total X̃ signal is the physically meaningful quantity. In this case, where the *C*_*3*_ axis of the pulse does not coincide with the C_3_ axis of the molecule, the 3N ± 1 selection rule is no longer enforced: 3N harmonics appear in the total spectrum. The dependence of the HHG yield on the geometric displacement is detailed in Figure S3. To provide a quantitative insight, in Table 1 we report the intensity ratio between the 3N and 3N ± 1 harmonic peaks for the asymmetrically stretched configuration in the pulse-molecule configuration shown in Figure 2, where the molecular C_3_ rotational axis does not coincide with the field C_3_ axis. We compute this ratio by averaging the intensities of the 3N ± 1 and 3N-order harmonics in the region 4H–15H. Although we show the HHG spectra on a logarithmic scale throughout this work, we report the 3N/(3N ± 1) ratio computed from the linear (nonlogarithmic) HHG yield. This ratio is roughly preserved between the total spectrum and channel A. By contrast, the ratio is strongly enhanced in the X′ and X̃ channel contributions, where the 3N and 3N ± 1 harmonics reach comparable intensities. The interpretation is that X′ and X̃, individually, carry a strong imprint of the broken symmetry due to the directionality of MOs, which is then softened when the two channels dynamically interact. In contrast, for the equilibrium geometry with a molecule-pulse configuration where the rotational symmetry of the pulse coincides with the molecular symmetry, the ratio 3N/(3N ± 1) drops to 4.8 × 10^–5^ for the total spectrum and remains strictly below 1.5 × 10^–4^ for both channel contributions. Similarly, for the rotationally averaged spectrum shown in the bottom panel of Figure 2, this ratio is 2.1 × 10^–4^.

**Table 1:** Intensity Ratio between 3N and 3N ± 1 Harmonic Signals for Different Channel Contributions, for the C_1_ Projection of the Asymmetrically Stretched Configuration with Maximum Elongation of 4 × 10^–3^ Å

HHG signal	3*N*/(3*N* ± 1)
Total	4.2 × 10^–3^
X′	4.6 × 10^–1^
X̃	9.6 × 10^–1^
X′ + X̃	3.6 × 10^–3^
A	6.0 × 10^–3^

**Figure 4:**
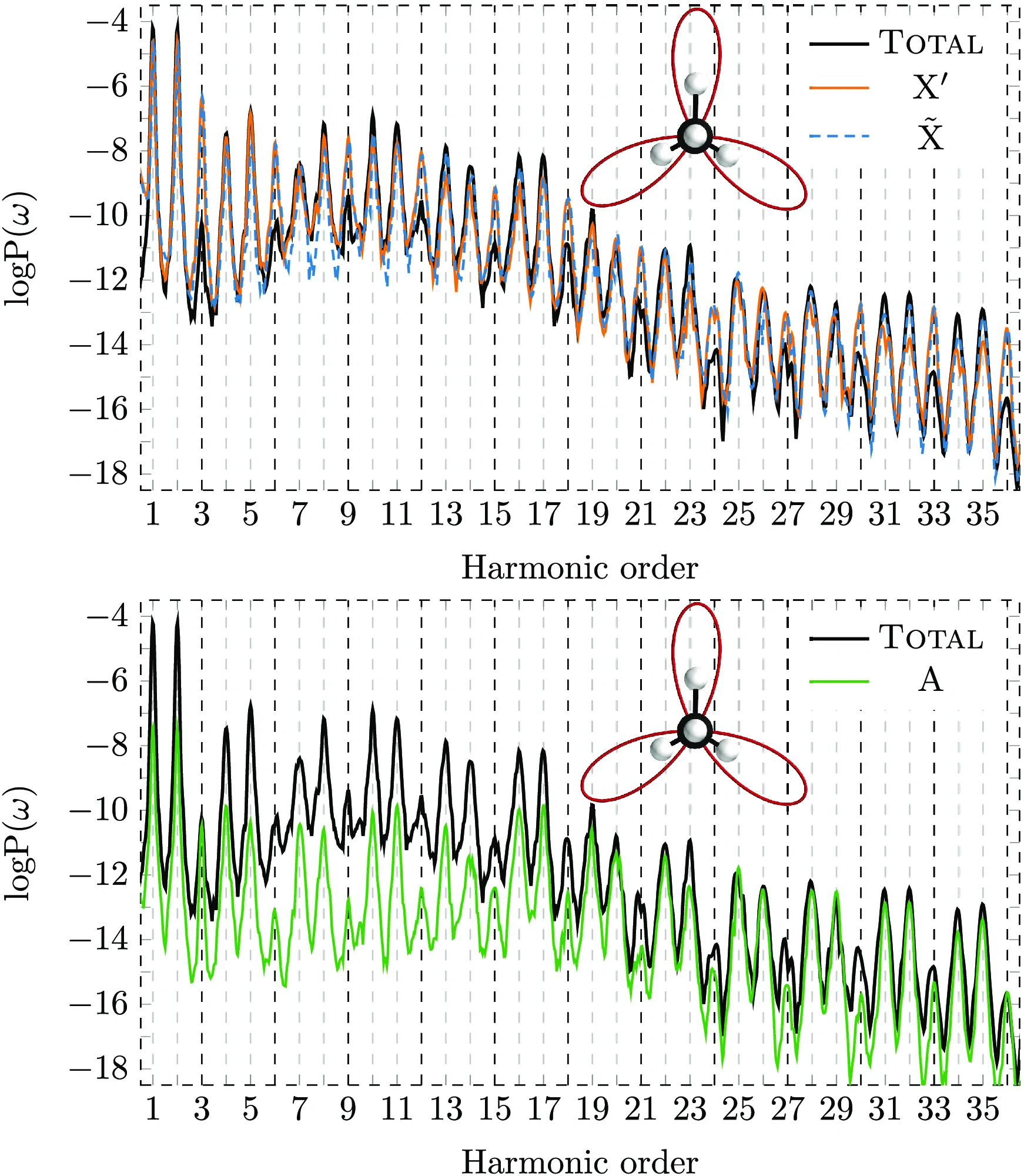
Top panel: MO decomposition of the CH_4_ HHG spectrum in the asymmetrically stretched geometry with a C_1_ symmetry projection on the pulse polarization plane, displaying the total spectrum and the two quasi-degenerate X′ and X̃ channel contributions. Bottom panel: the total spectrum and the A contribution.

Indeed, summing the contributions of the X′ and X̃ channels, the resulting spectrum nearly overlaps with the total spectrum, i.e. with reduced intensity of 3N harmonics, as shown in the upper panel of Figure 5. Only minor differences are observed beyond the cutoff region, where the A channel becomes significant. The recovered intensity ratio arises from destructive interference between the two channels for the 3N harmonics and predominantly constructive interference for the 3N ± 1 harmonics (bottom panel of Figure 5).

**Figure 5:**
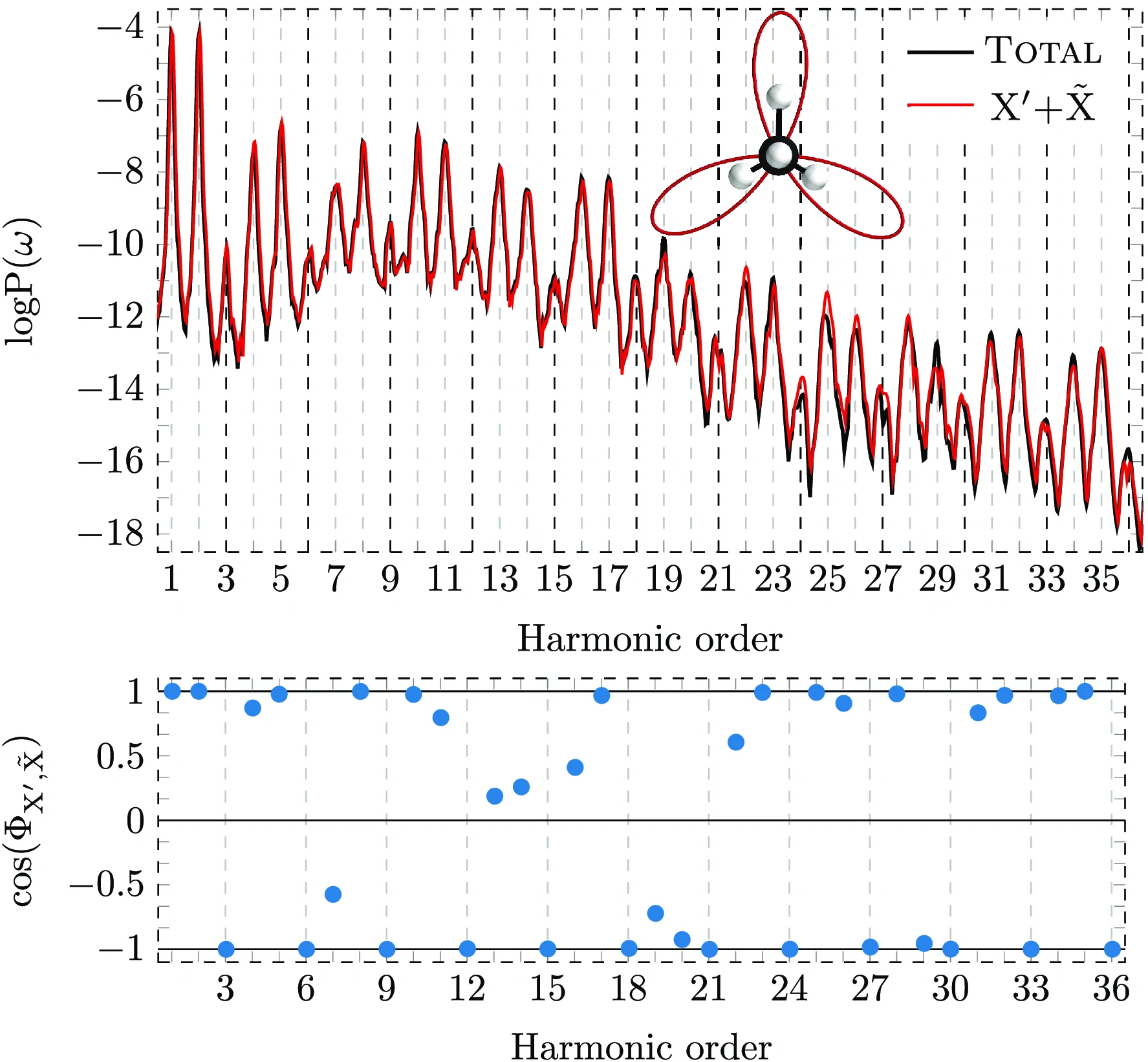
Top panel: MO decomposition of the CH_4_ HHG spectrum in the asymmetrically stretched geometry with a C_1_ symmetry projection on the pulse polarization plane, displaying the total spectrum and the two quasi-degenerate X′ and X̃ channel contributions summed together. Bottom panel: interferences between the X′ and X̃ channels.

Unlike the equilibrium decomposition, where the appearance of forbidden 3N harmonics in X_A,B,C_ contributions originates from the arbitrary representation of the degenerate X channel, the X′ and X̃ channels correspond to two distinct quasi-degenerate ionization channels. Consequently, the interference responsible for recovering the correct 3N/(3N ± 1) intensity ratio in the X′ + X̃ spectrum reflects the physical interaction between the two quasi-degenerate cationic states generated by the asymmetric distortion of the tetrahedral geometry.

To validate our approach, we also considered the selection rules when the asymmetrically stretched CH_4_ is oriented such that the only different C–H bond is perpendicular to the polarization plane, thus restoring C_3_ symmetry. The spectra for the asymmetrically stretched configuration with C_1_- and C_3_-symmetric projections are compared in Figure S4, using the same displacement as in Figure 1(c) (one CH bond elongated by 4 × 10^–3^ Å, the other three shortened by 4/3 × 10^–3^ Å). The MO decomposition for the C_3_ projection is presented in Figure S5. In Figure S6, the MO decomposition is reported for a larger bond elongation of 6 × 10^–3^ Å (compression of 2 × 10^–3^ Å on the other three bonds), used for illustration purposes only.

Since tetrahedral symmetry is maintained in the symmetrically stretched configuration, the HHG spectra retain the 3N ± 1 selection rule (Figure 2). The only spectral differences relative to equilibrium consist of minor variations in harmonic yield. As the molecular symmetry and HOMO degeneracy are preserved, the MO decomposition follows the same analysis as in the equilibrium case (Figures S7–S9).

In our simulations, we employ a driving pulse with 30 optical cycles (with period ∼2.67 fs) to achieve the spectral resolution required to resolve individual harmonic orders. The resulting HHG spectrum is therefore a coherent average over many emission events (∼90), each occurring on an extremely short time scale. While this approach is optimal for revealing symmetry-imposed selection rules, tracking vibrational motion in a pump–probe experiment would require a few-cycle or gated probe pulse to isolate a single attosecond burst, at the expense of spectral resolution. This trade-off is inherent to the technique and must be taken into account in the context of experimental implementations. The effect of the number of optical cycles on the HHG spectrum is reported in Figure S10 of the SI. The distorted geometries studied in this work are not, in and of themselves, a representation of nuclear motion in the ground or excited vibrational state. This motivates the use of an IR pump in the proposed experimental scheme. The expected value of the molecular geometry in the vibrational ground state coincides with the tetrahedral equilibrium structure, because the ground-state nuclear wave function samples displacements symmetrically: this leads to destructive interference and cancellation of the 3N signal. We verified this effect by considering two geometries distorted by the inverted displacement: by coherently summing the corresponding dipoles with equal weight, we obtain an HHG spectrum with suppression of the 3N harmonics (Figure S11 and related discussion in the SI). In contrast, the IR pump is essential to create a population imbalance. The IR pump creates a coherent vibrational wavepacket that is preferentially localized on one side of the potential energy curve, lifting the destructive interference and allowing the 3N signal to emerge: that is, it would mean summing the dipoles of the two structures with different weights.

A practical route to observing the symmetry-forbidden 3N harmonics in vibrationally excited molecules is a differential measurement comparing the HHG spectrum with and without the IR pump. In the pump-off case, zero-point motion (ZPM) along the A_1_ and T_2_ modes provides a small but nonzero displacement around equilibrium but, as discussed, no 3N harmonics are expected on average.

In this Letter, we show how the sensitivity of the ω/2ω BCR field to the rotational symmetry of the emitter allows imprinting structural effects of asymmetric normal modes on HHG spectra. Specifically, symmetry breaking associated with asymmetric modes lifts the selection rules, triggering emission of formally forbidden harmonics. In particular: (i) the ω/2ω BCR pulse enforces a 3N ± 1 selection rule dictated by the C_3_ dynamical symmetry of the pulse–molecule system; (ii) this rule is broken by asymmetric nuclear distortions, generating forbidden 3N harmonics whose intensity scales with the degree of distortion; (iii) this establishes, in principle, a route to probe asymmetric nuclear motion via HHG, of potential use in a pump–probe scheme, provided molecular alignment/orientational coherence and an IR pump are achieved, since the IR pump generates an imbalance in the conformational sampling. In an aligned ensemble of emitters, this process is synchronized across all molecules, provided the IR pump is shorter than the vibrational period, as discussed before.

Aligning methane is practically not feasible because its high tetrahedral symmetry results in neither a permanent dipole moment nor a significant anisotropy of the polarizability tensor. A possible route is the use of lower-symmetry molecules, such as fluoromethane (CH_3_F), which are more amenable to laser alignment and have similar vibrational periods. In the pioneering work of ref 70, the authors show that the nature of forbidden harmonics in gas-phase SF_6_ is due to symmetry-breaking normal modes.

Since surface adsorption naturally enforces a fixed molecular orientation, extending HHG spectroscopy to molecules adsorbed on surfaces becomes an effective approach for this type of investigation. Future developments may also include the investigation of vibrational modes in aligned organic molecular crystals, whose HHG spectra have recently been shown to encode information about both the crystal structure and the constituent molecules.^69^ Extending our TD-CIS scheme to such systems could be achieved either by incorporating the environment via a hybrid QM/MM framework (thus practically retaining a single-emitter model) or by simulating HHG spectra in a molecular dimer as a minimal multiemitter prototype. The study of these systems offers a promising route for extending the present concepts to more complex condensed-phase environments.

## Supplementary Material



## Data Availability

Data are available from the corresponding author upon reasonable request.

## Notes

Theoretical approach and computational details, harmonic polarization, effect of ionization parameters., harmonic intensity as a function of the spatial displacement, C_1_/C_3_ projection comparison and MO decomposition for the asymmetric-stretched methane, HHG spectra of symmetric-stretched methane, role of the number of optical cycles, and role of the sign of the displacements (PDF)
