# Supplementary material for: Symmetry and Vibrational Control of Multi-Channel HHG in Methane
Source: J Phys Chem Lett. 2026 Oct 8;17(43):12303–9. doi: 10.1021/acs.jpclett.6c02522 (PMC13726472; doi:10.1021/acs.jpclett.6c02522)
Supplement: Supplementary file 1 [file jz6c02522_si_001.pdf]

Supporting Information for "Symmetry and Vibrational Control of Multi-Channel HHG in Methane"

Marco Marchetta,† Eleonora Luppi,‡ and Emanuele Coccia*,†

†*Dipartimento di Scienze Chimiche e Farmaceutiche, Università di Trieste, via L. Giorgieri 1, 34127, Trieste, Italy*

‡ *Laboratoire de Chimie Théorique, Sorbonne Université, CNRS, Paris, F-75005, France*

E-mail: ecoccia@units.it

Theoretical method

Real-Time propagation

Electron dynamics in a spectroscopic target irradiated by an external time-dependent electric field is described by the time-dependent Schrödinger equation (TDSE)

$$i\frac{\partial|\Psi(t)\rangle}{\partial t} = \left(\hat{H}_0 + \hat{V}(t)\right)|\Psi(t)\rangle, \tag{1}$$

where $\hat{H}_0$ is the time-independent field-free molecular electronic Hamiltonian and $\hat{V}(t)$ is the time-dependent potential describing the interaction between the molecule and the external field. In the semi-classical dipole approximation, this interaction between the molecule and

the time-dependent external laser field can be written in the length gauge as

$$\hat{V}(t) = -\hat{\boldsymbol{\mu}} \cdot \mathbf{E}(t), \tag{2}$$

where $\hat{\boldsymbol{\mu}}$ is the molecular dipole operator and $\mathbf{E}(t)$ is the external electric field of the laser pulse.

In the RT-TD-CIS method, Eq. 1 is numerically approached by expanding the time-dependent wavefunction $|\Psi(t)\rangle$ in the space of the $\hat{H}_0$ eigenstates, i.e. the Hartree-Fock (HF) ground state ($k = 0$) and the excited states ($k > 0$)[1]

$$|\Psi(t)\rangle = c_0(t)|\psi_0\rangle + \sum_{k>0} c_k(t)|\psi_k\rangle, \tag{3}$$

where $c_k(t)$ are complex time-dependent coefficients and for $k > 0$

$$|\psi_k\rangle = \sum_{i}^{\text{occ}} \sum_{a}^{\text{vir}} r_{i,k}^{a} |\phi_i^a\rangle, \tag{4}$$

which contains singly-excited Slater determinants derived from the HF ground state $|\psi_0\rangle$, by exciting an electron from the occupied MO i to the virtual MO a.

The TDSE in Eq. 1 is thus propagated in the space of electronic states from Eq. 3

$$i\frac{\partial \mathbf{c}(t)}{\partial t} = \left(\mathbf{H}_0 + \mathbf{V}(t)\right)\mathbf{c}(t), \tag{5}$$

where $\mathbf{c}(t)$ is the vector of the coefficients $c_k(t)$, $\mathbf{H}_0$ is the matrix representing the field-free Hamiltonian, with elements $\mathbf{H}_{0,lk}(t) = \langle\psi_l|\hat{H}_0|\psi_k\rangle$ and $\mathbf{V}(t)$ is the matrix representing the light-matter interaction, with elements $\mathbf{V}_{lk}(t) = -\mathbf{E}(t)\langle\psi_l|\hat{\boldsymbol{\mu}}|\psi_k\rangle$. Further details are found in Refs. 2,3.

Once the time-dependent wavefunction $|\Psi(t)\rangle$ is determined, the time-dependent dipole

moment is computed as

$$\boldsymbol{\mu}(t) = \langle \Psi(t)|\hat{\boldsymbol{\mu}}|\Psi(t)\rangle = \sum_{lk} c_l^*(t) c_k(t) \langle \psi_l|\hat{\boldsymbol{\mu}}|\psi_k\rangle. \tag{6}$$

Finally, the HHG power spectrum is then obtained by applying the Fourier transform to $\boldsymbol{\mu}(t)$

$$P(\omega) = \left| \frac{1}{t_\mathrm{f} - t_\mathrm{i}} \int_{t_\mathrm{i}}^{t_\mathrm{f}} W(t) \langle \Psi(t)|\hat{\boldsymbol{\mu}}(t) \cdot \mathbf{n}_{\alpha'}|\Psi(t)\rangle e^{-i\omega t} dt \right|^2, \tag{7}$$

where t_i and t_f are the initial and final propagation times, $\mathbf{n}_{\alpha'}$ is the unit vector defining the direction of detection of the emitted signal and $W(t)$ is a window function.

MO decomposition of the HHG signal

For a singly-excited ansatz wavefunction, the time-dependent dipole can be further analyzed by decomposing it into the sum of occupied MO contributions[3–6]

$$\boldsymbol{\mu}(t) = \sum_i^{\mathrm{occ}} \boldsymbol{\mu}_i(t), \tag{8}$$

where the contribution of the ith occupied orbital is computed as

$$\begin{aligned} \boldsymbol{\mu}_i(t) &= |c_0(t)|^2 \langle i|\hat{\boldsymbol{\mu}}|i\rangle \\ &+ \sum_{k>0} \sum_a^{\mathrm{vir}} \left(c_0^*(t) c_k(t) + c_k^*(t) c_0(t) \right) r_{i,k}^a \langle i|\hat{\boldsymbol{\mu}}|a\rangle \\ &+ \sum_{k>0} \sum_{l>0} \sum_j^{\mathrm{occ}} \sum_a^{\mathrm{vir}} c_l^*(t) c_k(t) r_{i,l}^a r_{i,k}^a \langle j|\hat{\boldsymbol{\mu}}|j\rangle \\ &+ \sum_{k>0} \sum_{l>0} \sum_a^{\mathrm{vir}} \sum_b^{\mathrm{vir}} c_l^*(t) c_k(t) r_{i,l}^a r_{i,k}^b \langle a|\hat{\boldsymbol{\mu}}|b\rangle \\ &- \sum_{k>0} \sum_{l>0} \sum_j^{\mathrm{occ}} \sum_a^{\mathrm{vir}} c_l^*(t) c_k(t) r_{i,l}^a r_{j,k}^a \langle j|\hat{\boldsymbol{\mu}}|i\rangle. \end{aligned} \tag{9}$$

By inserting the MO contribution $\boldsymbol{\mu}_i(t)$ in Eq. 7 one can compute the partial HHG

spectrum coming from the i-th MO as

$$P_i(\omega) = \left| \frac{1}{t_\mathrm{f} - t_\mathrm{i}} \int_{t_\mathrm{i}}^{t_\mathrm{f}} W(t) \boldsymbol{\mu}_i(t) \cdot \mathbf{n}_{\alpha'} e^{-i\omega t} dt \right|^2. \tag{10}$$

Moreover, by using the quantity $p_i(\omega)$ given by

$$p_i(\omega) = \frac{1}{t_\mathrm{f} - t_\mathrm{i}} \int_{t_\mathrm{i}}^{t_\mathrm{f}} W(t) \boldsymbol{\mu}_i(t) \cdot \mathbf{n}_{\alpha'} e^{-i\omega t} dt, \tag{11}$$

the relative phase $\Phi_{i,j}$ between contributions to the total HHG spectrum from the different MOs i and j is computed

$$\cos(\Phi_{i,j}) = \mathrm{Re} \left(\frac{p_i^*(\omega) p_j(\omega)}{|p_i(\omega)||p_j(\omega)|} \right). \tag{12}$$

Harmonic helicity

The C_3 dynamical symmetry of $\omega/2\omega$ BCR pulses dictates the emission of circularly polarized 3N$\pm$1 harmonics with alternating ellipticities, confined within the polarization plane of the driving field. While linearly polarized 3N harmonics may, in principle, be emitted orthogonal to the field polarization plane in systems without inversion symmetry.[7] Harmonic helicity, in a given $\alpha\beta$ plane, can be computed as

$$\varepsilon = \frac{|d_+(\omega)| - |d_-(\omega)|}{|d_+(\omega)| + |d_-(\omega)|}, \tag{13}$$

where

$$d_\pm(\omega) = (d_\alpha(\omega) \pm i d_\beta(\omega))/\sqrt{2}, \tag{14}$$

and $d_\alpha(\omega)$($d_\beta(\omega)$) is the harmonic intensity at frequency ω along the α (β) direction. In Figure S1 we report the spectra emitted along a direction contained in the polarization plane and orthogonal to it, together with the helicity.

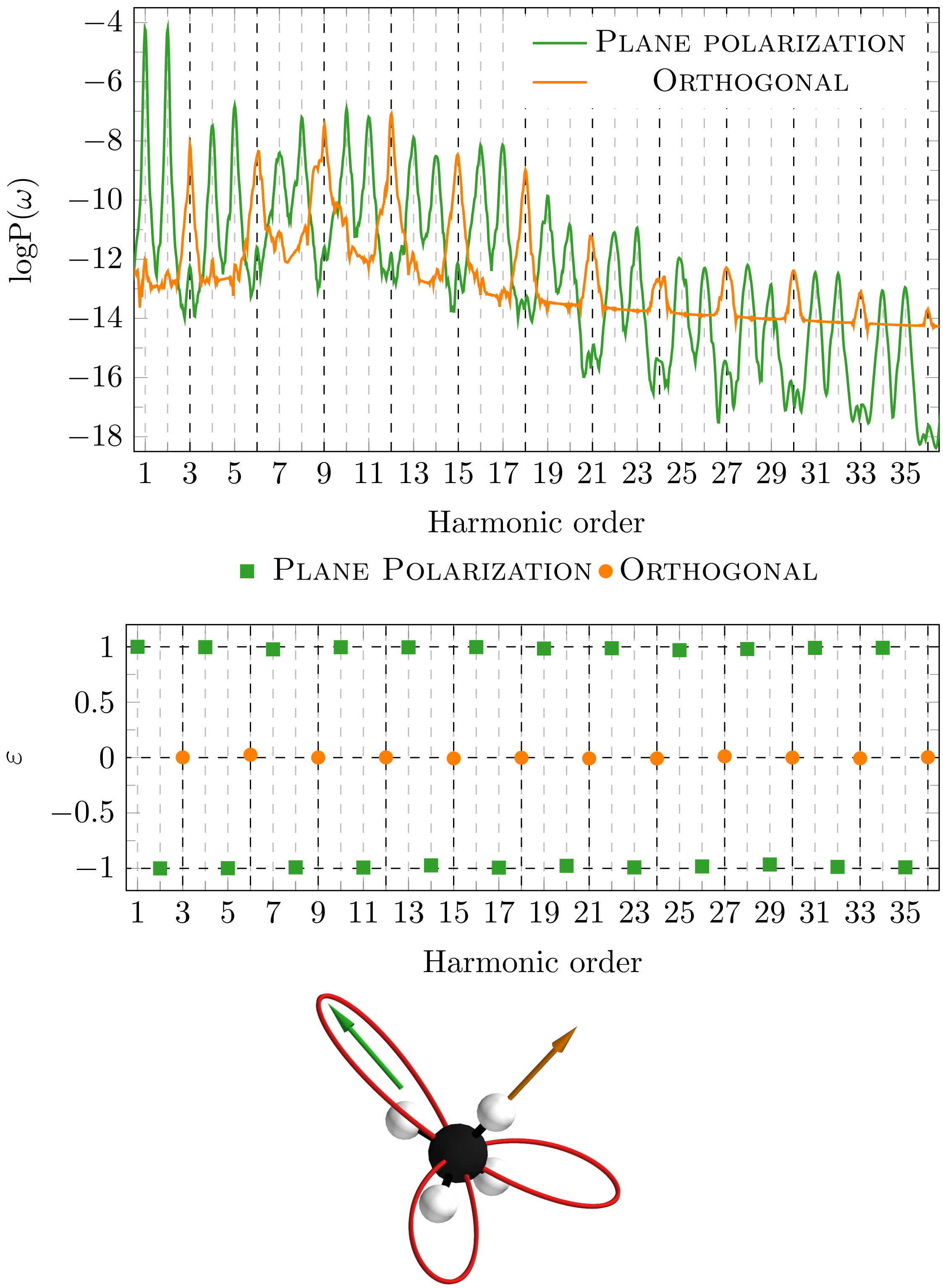

Figure S1: The emitted harmonics have different polarization properties: 3N±1 order harmonics are circularly polarized with alternating ellipticities (± 1) in the pulse polarization plane, while 3N linearly polarized harmonics are emitted orthogonally to the pulse polarization plane. Upper panel: emitted spectra in the plane polarization and orthogonal to it. Middle panel: ellipticities of the emitted harmonics. Bottom panel: representation of the molecule-pulse configuration and two directions in which emitted harmonics are recorded.

Computational details

The strong-field real-time simulations are carried out at fixed nuclear geometries. The equilibrium configuration consists of a tetrahedral geometry with a bond length of 1.08598 Å, obtained at the CCSD level of theory using an aug-cc-pV5Z basis set.[8] The symmetrically stretched configuration is computed by stretching all CH bonds by the same length (Δ), while the asymmetric configurations are obtained by elongating/shortening one bond by Δ and shortening/elongating the other three by $\Delta/3$. These geometries are then used in subsequent quantum chemistry simulations performed with the Q-chem software.[9]

To describe the time-dependent electron dynamics, CIS calculations are performed to extract the energies and transition dipole moments of the ground state and 805 excited states. These quantities are subsequently used as input for the Light code, which performs the real-time propagation of the time-dependent wave function under the influence of an external time-dependent electric field. The computational protocol we use to compute HHG spectra has proven accurate across a range of atomic and molecular systems.[2–5,10–12] Central to this approach is the construction of the basis set to properly describe both Rydberg and continuum states.[1,2,12–14] For the carbon atom, we use a fourfold augmented Dunning basis set,[15] 4aug-cc-pVTZ, supplemented with four optimized Gaussian continuum functions (K),[16] giving the 4aug-cc-pVTZ+4K basis set. In the same way, for the four hydrogen atoms, we constructed a 3aug-cc-pVDZ+3K basis set. In the present calculations, the basis set and the number of electronic states were chosen such that all the ionization channels contributing to the reported spectra lie within the energy window spanned by the electronic wavefunction.

For all HHG spectra simulated in this work, we employed a $\cos^2$-shaped pulse (see Eq. 1 of the main text) with a carrier frequency of $\omega = 0.057$ Ha (1.55 eV, 800 nm) and a peak intensity of $I = E_0^2/2 = 6.0 \times 10^{13}$ W/cm^2, . The total pulse duration is 30 optical cycles (o.c.), where 1 o.c. $= 2\pi/\omega_0$. The number of optical cycles governs the spectral resolution, which is critical for resolving the 3N harmonic orders. In Figure S10, we compare HHG spectra of the molecule at its equilibrium geometry with that of an asymmetrically

stretched (4×10^{-3} Å) configuration across various pulse durations. Although the signal at 3N orders for the asymmetrically stretched molecule is systematically higher than that of the equilibrium geometry, at least 20 o.c. are required to unambiguously detect 3N harmonics.

A discrete time step of 1.21 attoseconds (0.05 a.u.) was used for the propagation.

Displacement values used in this work ensure the accuracy of the electronic structure of the ground state, calculated at the HF level.

A further crucial aspect is the mitigation of artificial reflections and the treatment of basis set incompleteness in describing the outgoing electronic wavepacket. In this regard, we employ a heuristic lifetime model.[17] This approach consists of adding an imaginary term $-i\Gamma_k/2$ to the CIS energies E_k^{CIS}, where Γ_k represents the inverse lifetime of the excited state k above the ionization threshold:

$$\Gamma_k = \begin{cases} 0 & \text{if } (E_k^{CIS} - E_{\mathrm{HF}}) < I_p, \\ \sum_i \sum_a |r_{i,k}^a|^2 \frac{1}{d}\sqrt{\epsilon_a} & \text{if } (E_k^{CIS} - E_{\mathrm{HF}}) > I_p \text{ and } \ \epsilon_a > 0, \end{cases} \tag{15}$$

where $|r_{i,k}^a|^2$ is the excitation probability from orbital i to a, E_{HF} is the HF ground-state energy, d is the escape length of the electron during the lifetime $\tau_k = 1/\Gamma_k$, I_p is the ionization energy, ϵ_a the energi of the a–th virtual orbital. I_p was determined via Koopmans' theorem, resulting in $I_p = 14.83$ eV (HOMO) for the equilibrium geometry and $I_p = 14.80$ eV (HOMO) for the stretched configurations. In Figure S2 we compare the spectra obtained using two different values of d corresponding to the quiver radius, $d = E_0/\omega_0^2$, which corresponds to 12.5 a_0 and twice the quiver radius 25.0 a_0. Moreover, we compared the results using also a modified version of the heuristic model,[2,18] in which Γ_k is computed as:

$$\Gamma_k = \begin{cases} 0 & \text{if } (E_k^{CIS} - E_{HF}) < I_p, \\ \sum_i \sum_a |r_{i,k}^a|^2 \frac{1}{d_0}\sqrt{\epsilon_a} & \text{if } I_p < (E_k^{CIS} - E_{HF}) < E_c \text{ and } \epsilon_a > 0, \\ \sum_i \sum_a |r_{i,k}^a|^2 \frac{1}{d_1}\sqrt{\epsilon_a} & \text{if } (E_k^{CIS} - E_0) > E_c \text{ and } \ \epsilon_a > 0, \end{cases} \tag{16}$$

where E_c is the semiclassical cutoff energy (H17). Finally, a Lorentzian broadening was applied to all spectra reported in this work.

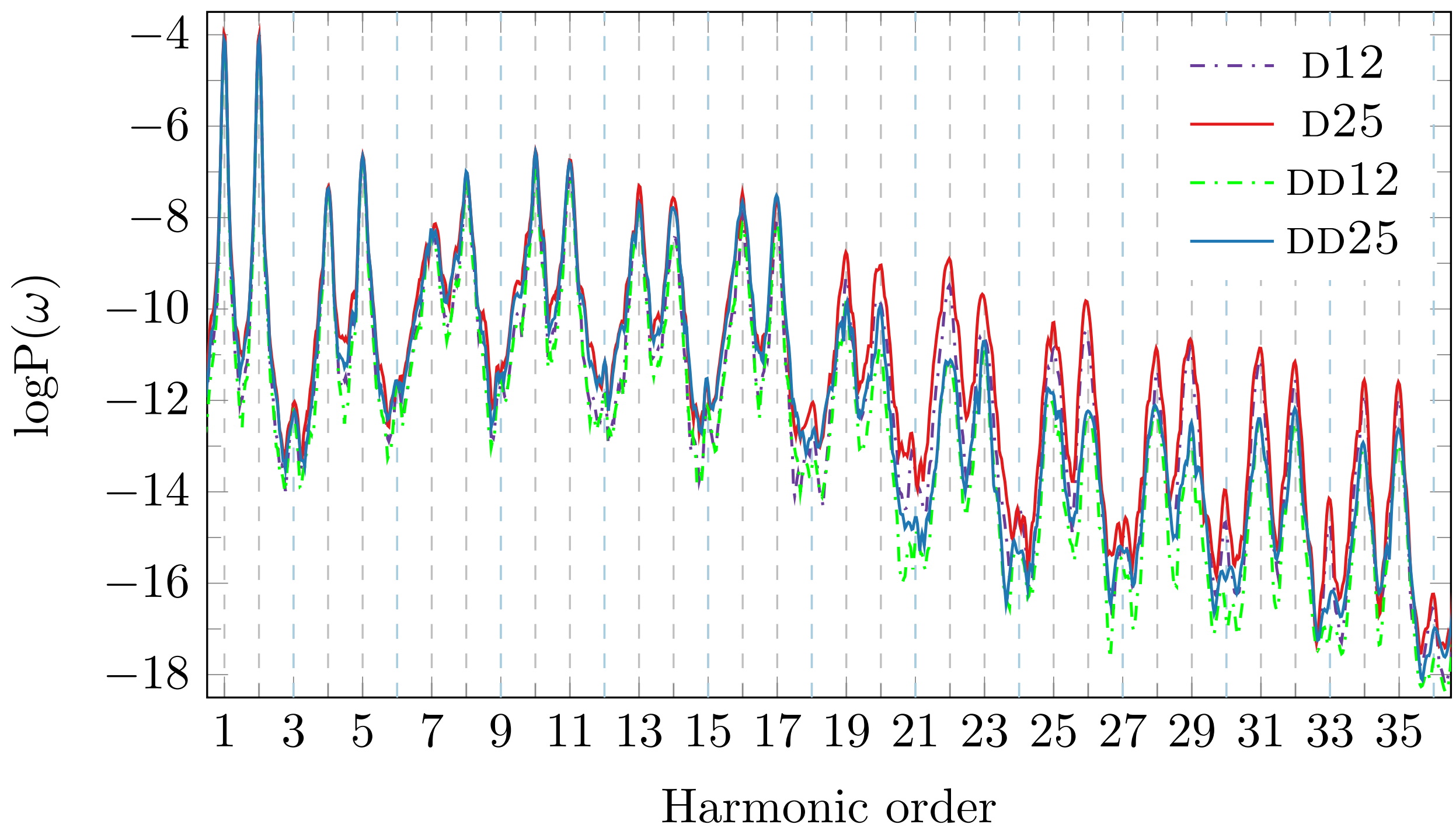

Figure S2: HHG spectra of CH_4 molecule obtained with the two different ionization models and different values of the parameter d. DX (DDX) refers to the ionization model in Eq. 15 (Eq. 16) with X being the value of d.

Effect of stretching on 3N harmonics

The symmetry-forbidden 3N harmonics are emitted in the asymmetric stretched configuration because the emitter symmetry no longer matches the pulse symmetry.

The intensity of the symmetry-forbidden 3N harmonics increases with the molecular asymmetry, and thus with the asymmetric stretching. The HHG spectra for various stretched geometries are presented in the upper panel of Figure S3. To evaluate the dependence of the HHG yield on the displacement from the equilibrium geometry, the yield for each stretched configuration is calculated as the average intensity of the 3N harmonics up to the 15th order. As shown in the bottom panel of Figure S3, this dependence exhibits a linear behavior.

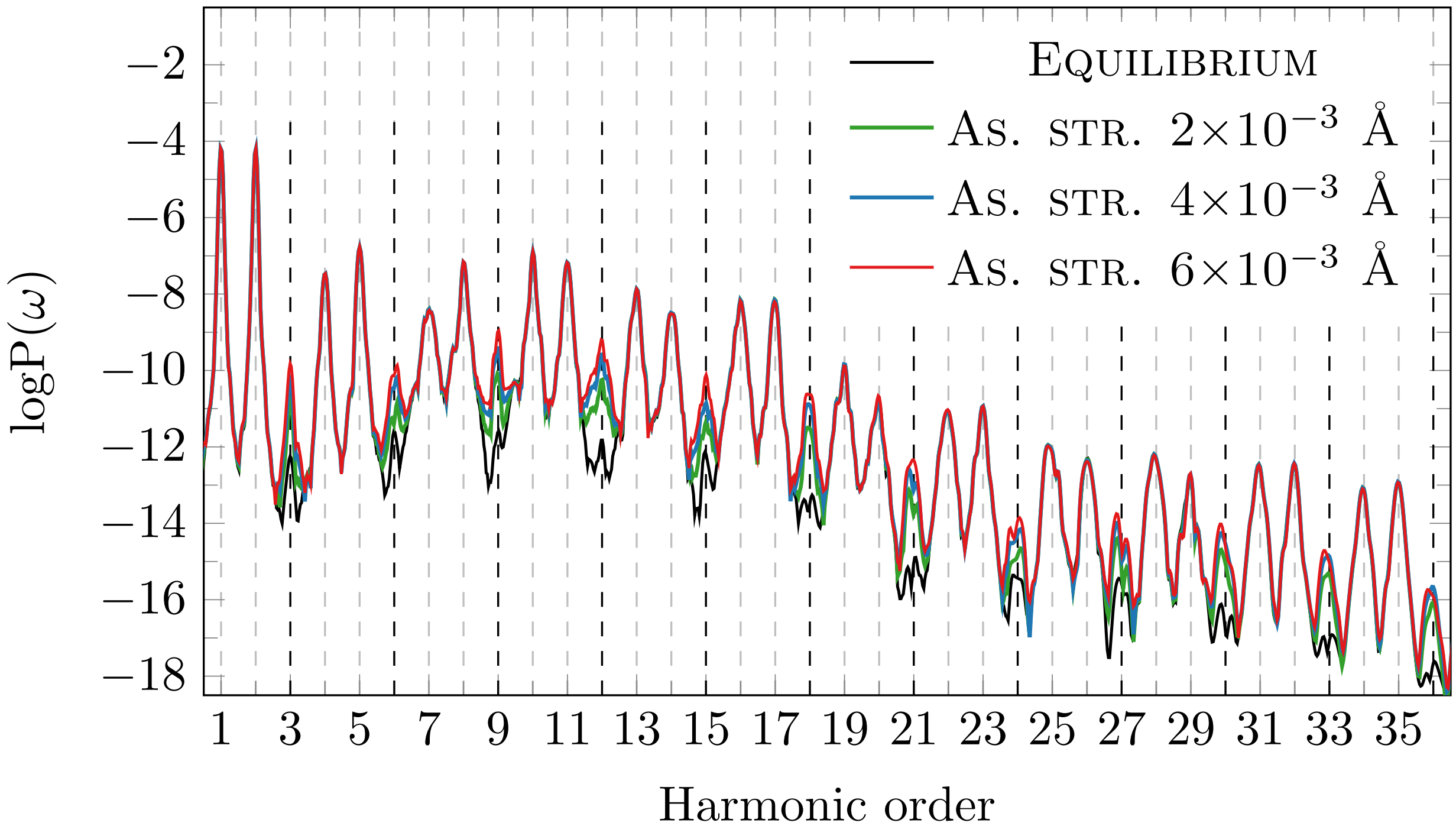

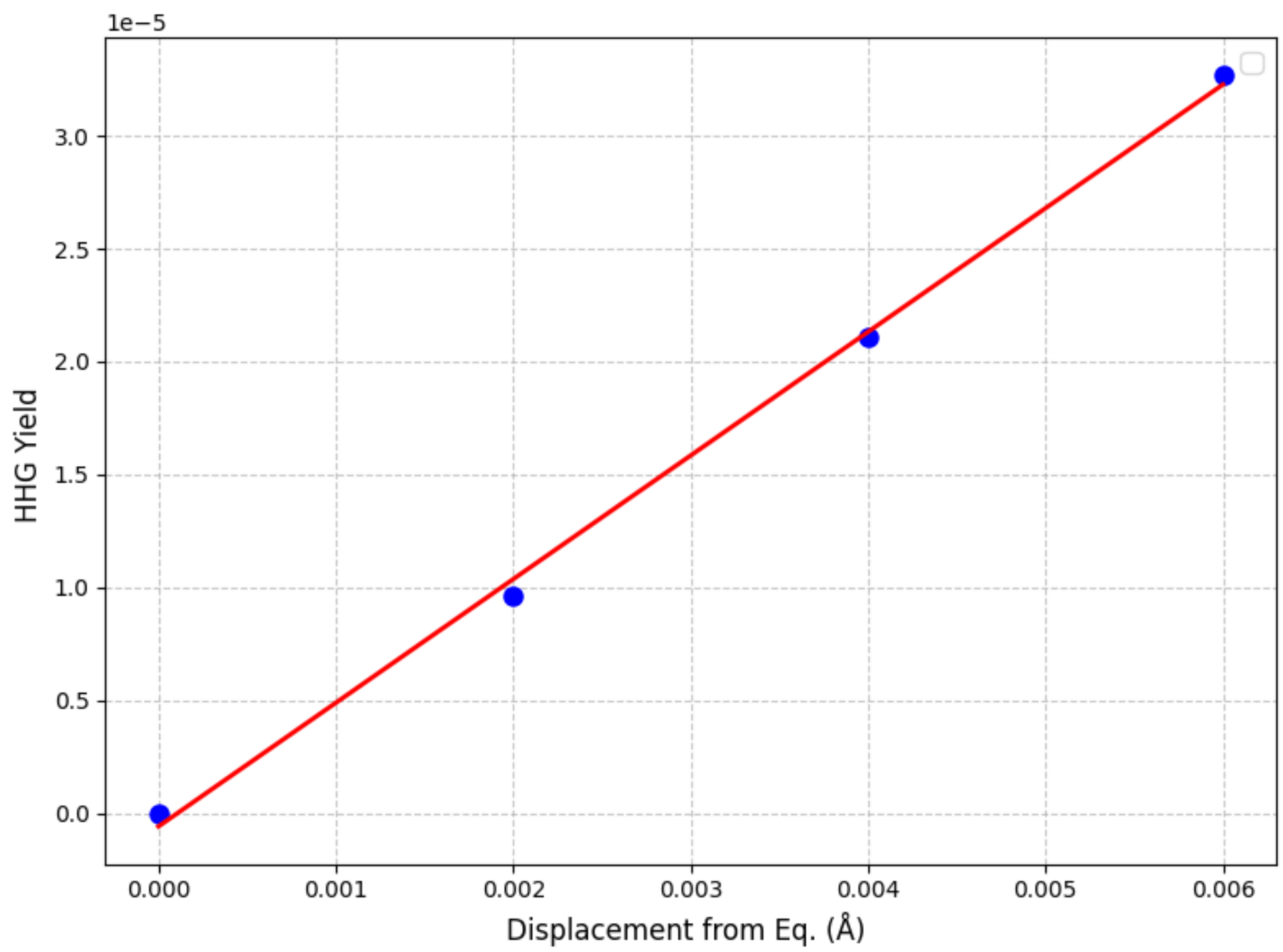

Figure S3: HHG spectra of CH_4 at the equilibrium configuration (black line) and asymmetrically stretched configuration, with one CH bond elongated by 2×10^{-3} Å and the other three shortened by $2/3\times10^{-3}$ Å (green line), elongated/shortened by $4\times10^{-3}/4/3\times10^{-3}$ Å (blue line), and by $6\times10^{-3}/2\times10^{-3}$ Å (red line), following the 3:1 elongation-to-compression ratio defined in the Computational details. Bottom panel: dependence of the 3N harmonics yield with respect to the displacement from equilibrium.

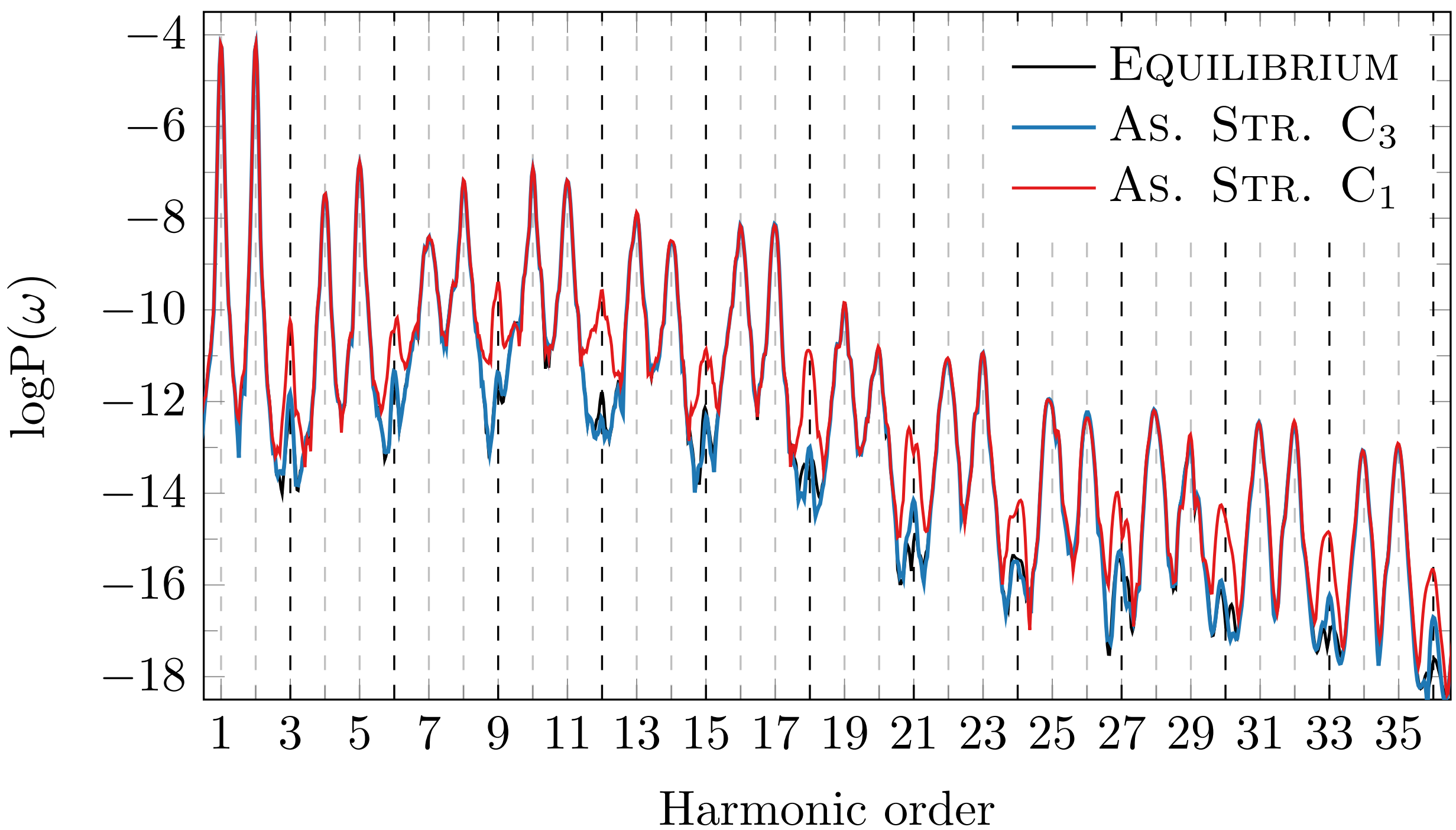

Figure S4: HHG spectra of CH_4 at the equilibrium configuration (black line) and asymmetrically stretched configuration, with the pulse that contains the C_3 projection of the stretched molecule (blue line) and the C_1 projection of the stretched molecule (red line).

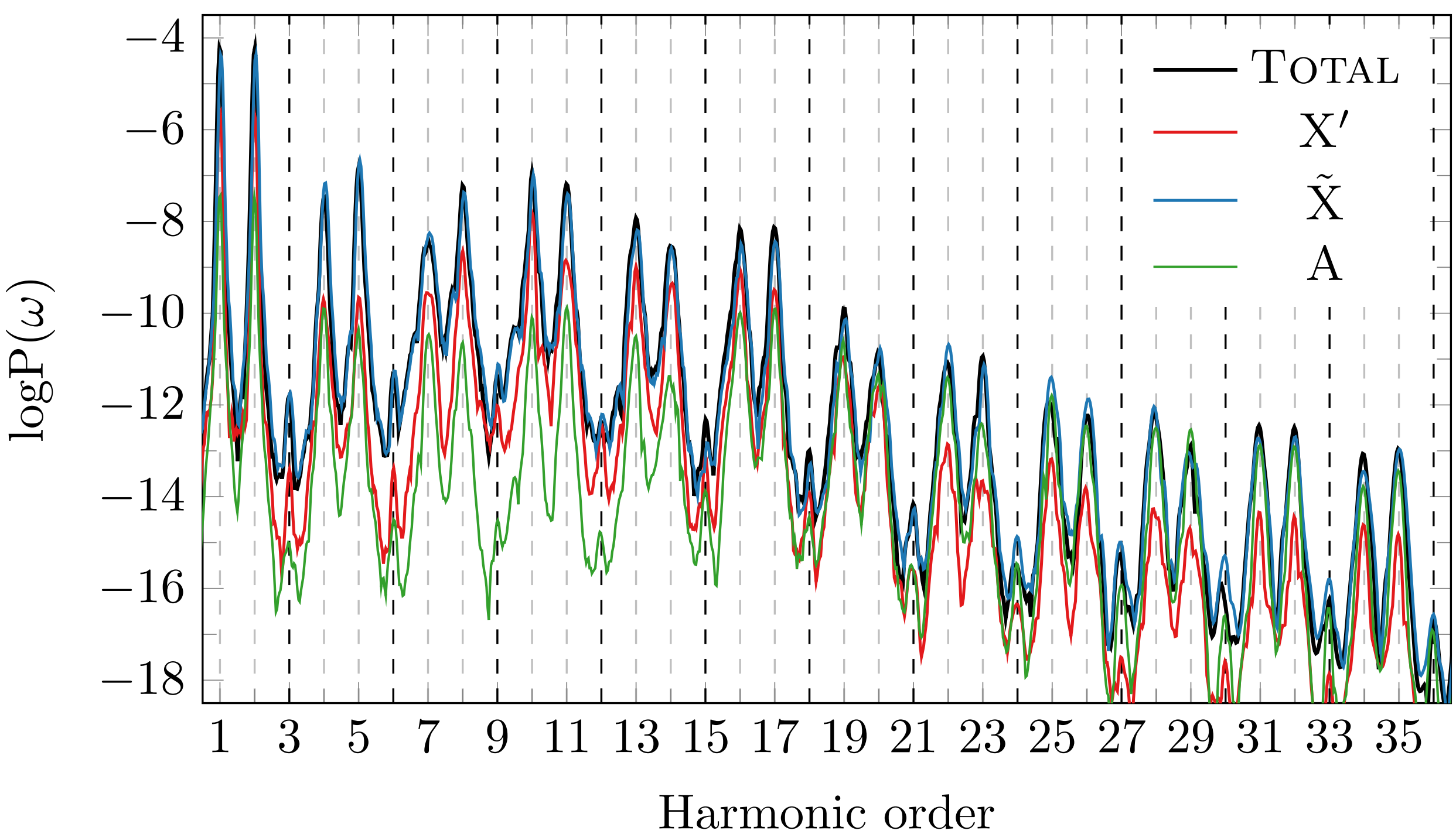

Figure S5: MO decomposition of HHG spectra of CH_4 at the asymmetric-stretched configuration (one CH bond elongated by 4×10^{-3} Å, the other three shortened by $4/3\times10^{-3}$ Å, as in Figure 1c of the main text) with pulse polarized perpendicularly to the elongated CH bond, so containing a C_3 projection. The degenerate $\tilde{X}$ contributions are summed together.

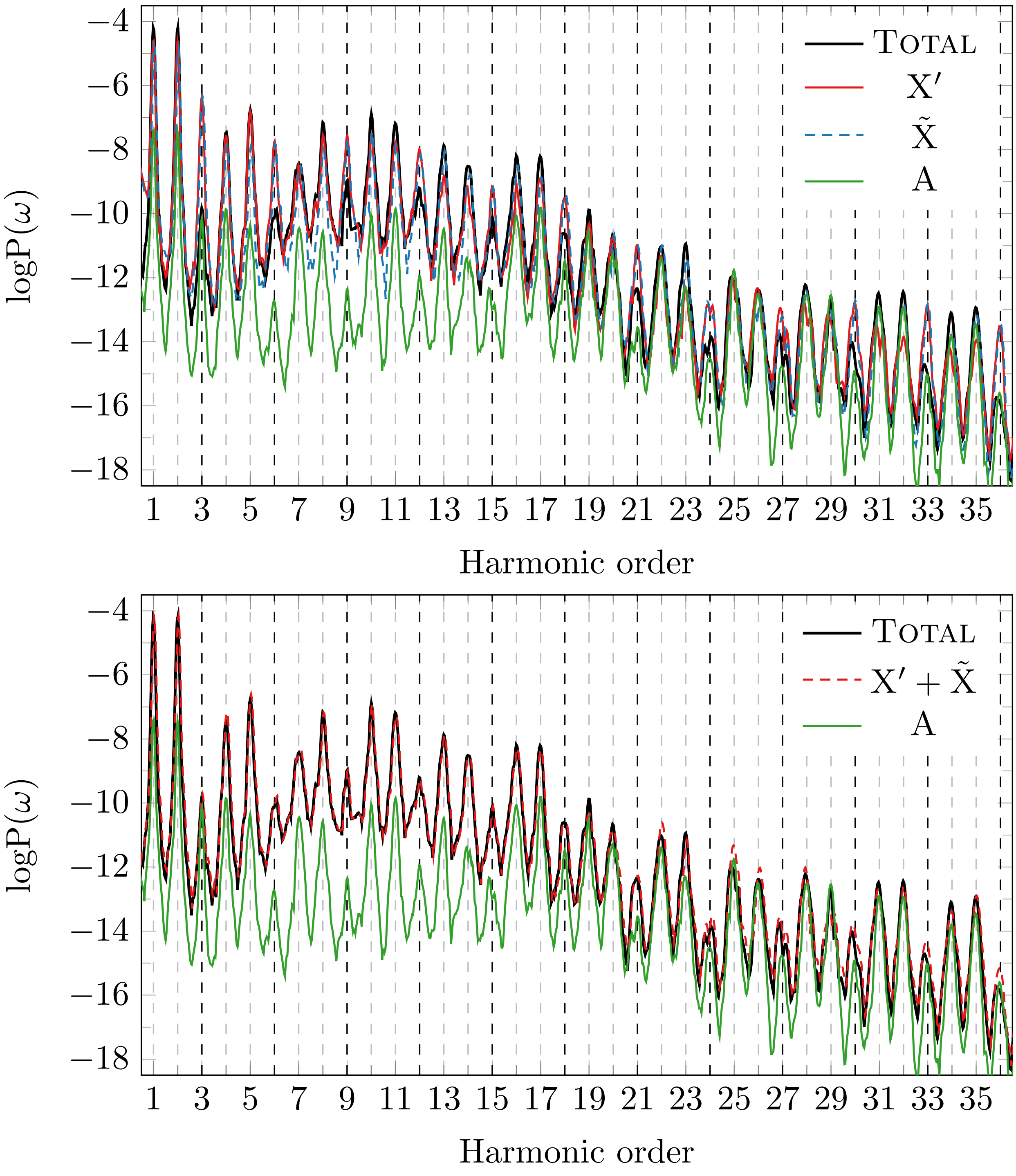

Figure S6: MO decomposition of HHG spectra of CH_4 at the asymmetric-stretched configuration with the elongated CH bond perpendicular to the polarization plane, with a maximum elongation of 6×10^{-3} Å (and a corresponding compression of 2×10^{-3} Å on the other three CH bonds). This displacement is larger than the one used in Figure S5 and in Figure 1c of the main text ($4\times10^{-3}/4/3\times10^{-3}$ Å); it is used here for illustration purposes only, analogous to Figure S3.

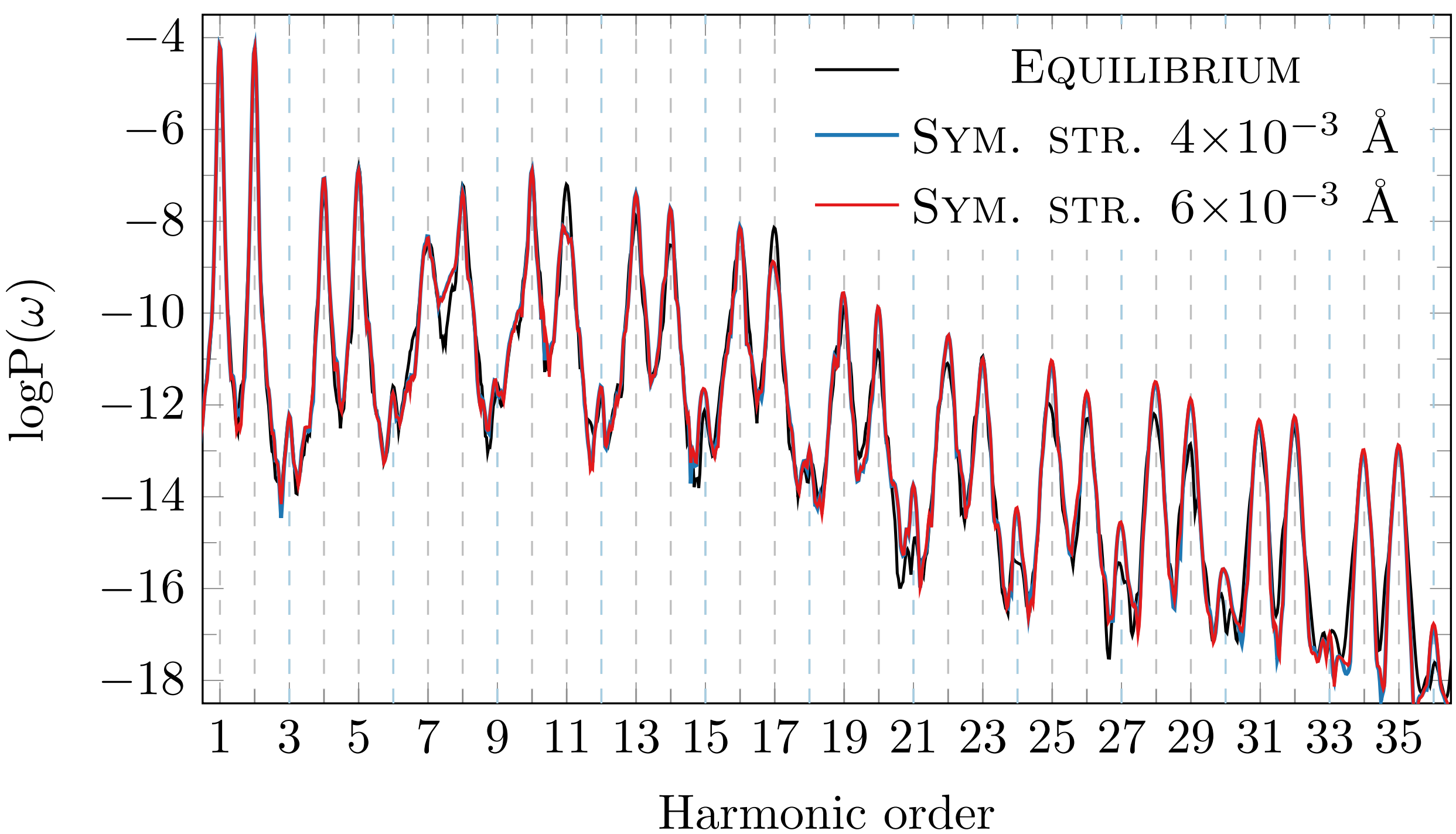

Figure S7: HHG spectra of CH_4 at the equilibrium configuration (black line) and symmetrically stretched configuration, with maximum elongation of 4×10^{-3} Å (blue line) and 6×10^{-3} Å (red line).

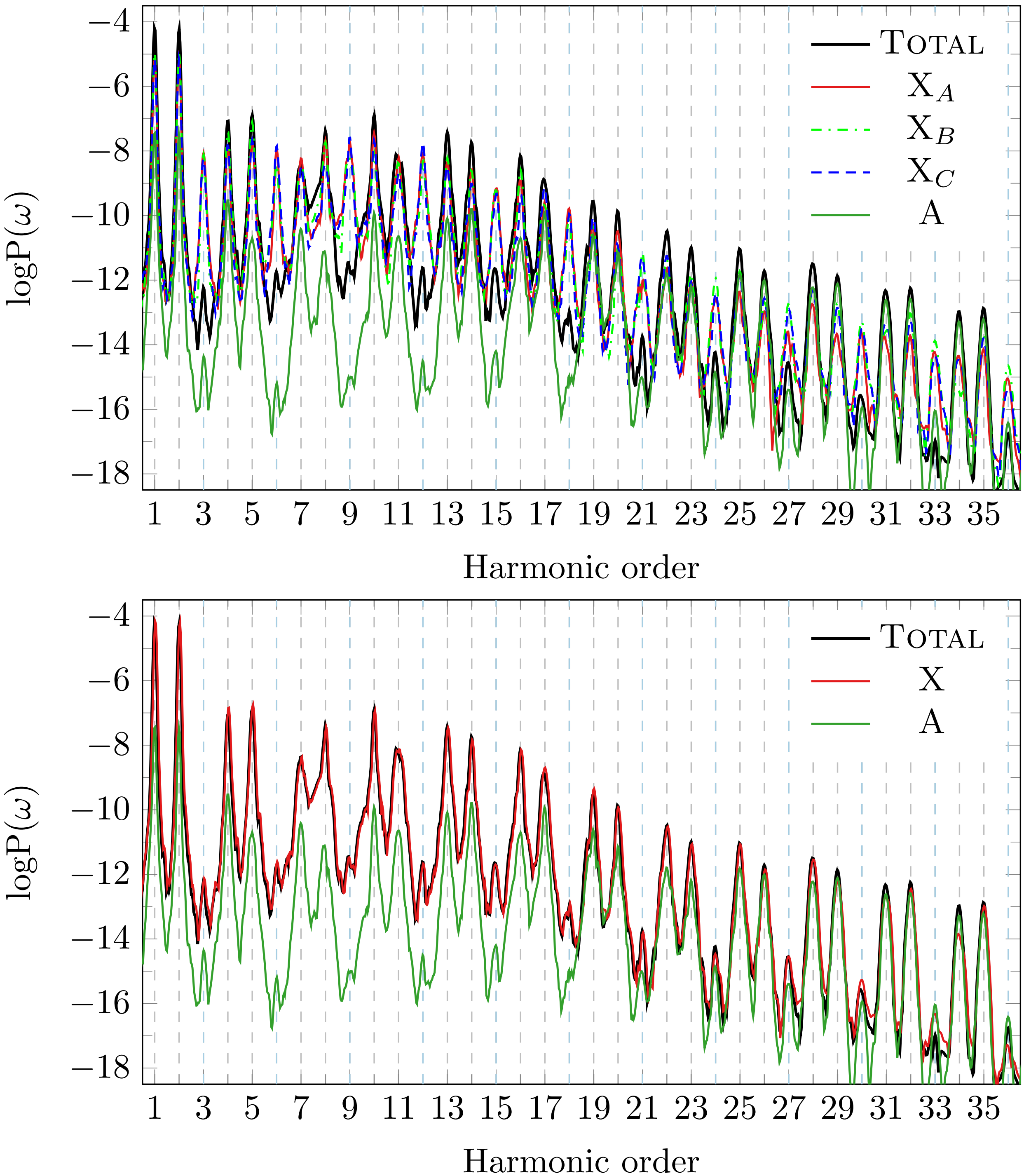

Figure S8: MO decomposition of HHG spectra of CH_4 at the symmetric stretched configuration, with bond stretching of 4×10^{-3} Å. Upper panel: the three X channels are shown separately. Lower panel: the three degenerate MO contributions to the X channel are summed together.

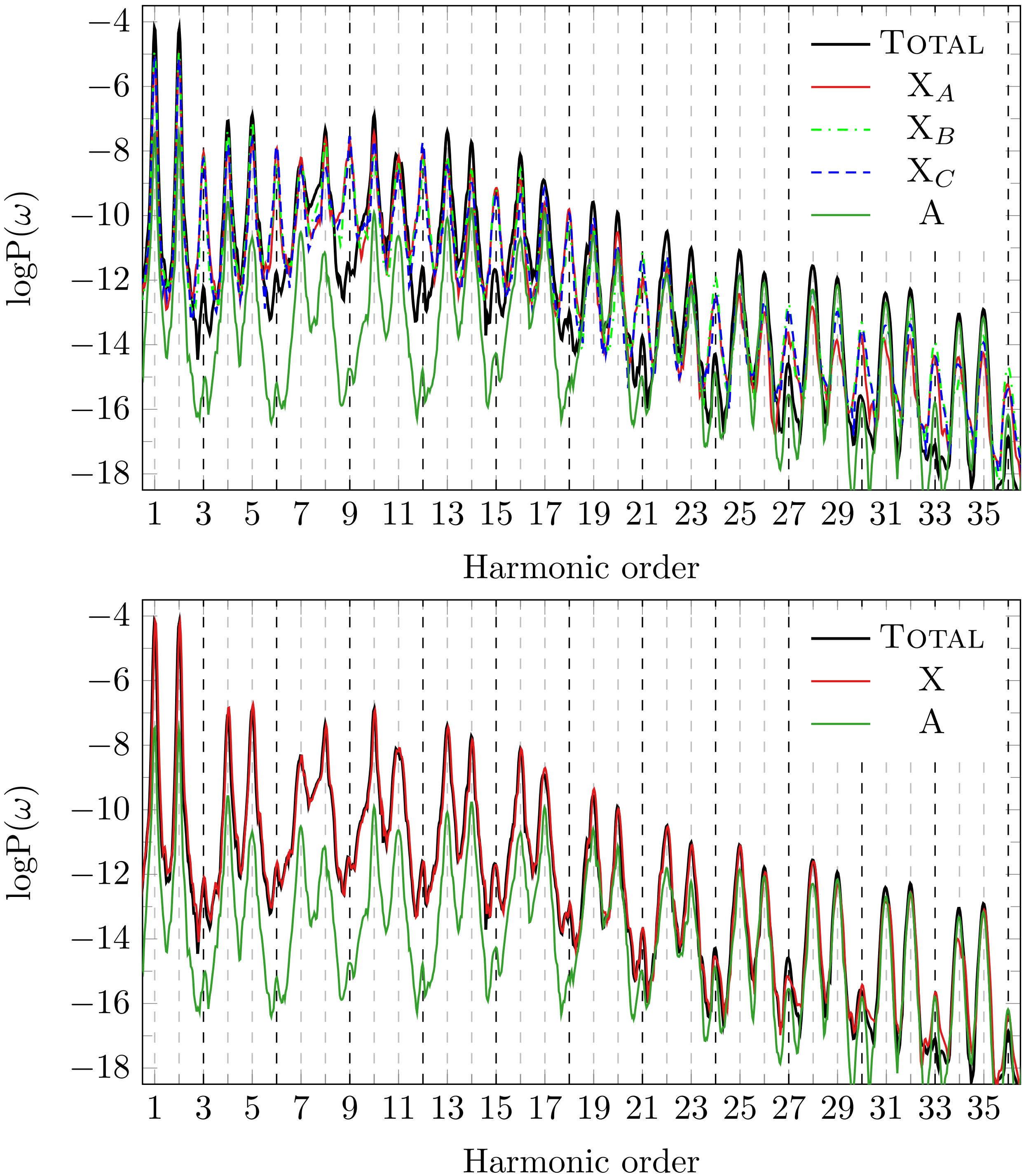

Figure S9: MO decomposition of HHG spectra of CH_4 at the symmetric stretched configuration, with bond stretching of 6×10^{-3} Å. Upper panel: the three X channels are shown separately. Lower panel: the three degenerate MO contributions to the X channel are summed together.

Combining P and N configurations

An asymmetrically stretched structure gives rise to 3N harmonics, as we observed by studying structures in which one bond is stretched by Δ while the remaining bonds are compressed by $\Delta/3$, where Δ is the displacement; we denote this structure as P. We found a comparable intensity of the 3N harmonics in the complementary case, in which one bond is compressed by Δ while the remaining bonds are stretched by $\Delta/3$; we indicate this structure as N. These two configurations are shown in Figure S11. The signals generated by structures P and N, together with the signal obtained by coherently summing the two time-dependent dipoles, are also shown in Figure S11. In the coherent sum of the two contributions, the 3N$\pm$1 harmonics are enhanced while the 3N harmonics are suppressed, even if not completely. This behavior is evident in the bottom panel of Figure S11, which shows the phase difference between the two signals: this phase difference results in constructive interference for the 3N$\pm$1 harmonics and destructive interference for the 3N harmonics. The HHG spectrum resulting from the Fourier transform of the coherent sum of P and N dipoles in Figure S11 is not intended to represent two spatially superimposed molecules. Rather, it represents the ensemble-averaged HHG signal expected from the vibrational ground state, where the nuclear wavefunction samples both complementary asymmetric configurations with equal probability and with a well-defined relative phase.

When the IR pump creates a coherent vibrational wavepacket, the wavefunction becomes preferentially localized on one side of the potential (a population imbalance between P and N), lifting the destructive interference and allowing the 3N signal to emerge. The magnitude of the 3N signal is therefore directly proportional to the population imbalance induced by the pump.

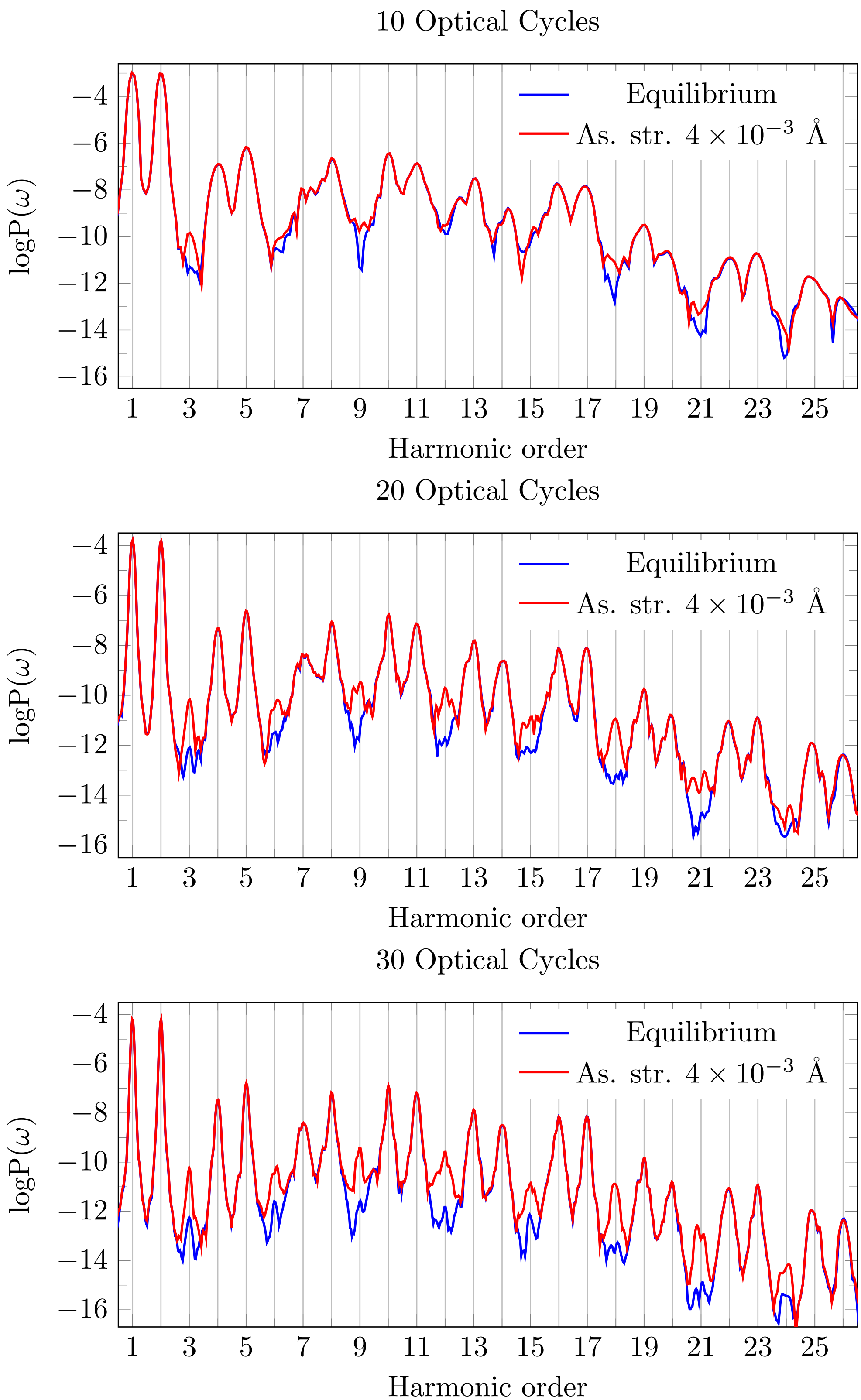

Figure S10: Dependence of the HHG spectral resolution on the laser pulse duration. HHG spectra are compared between the equilibrium geometry and an asymmetrically stretched molecular configuration for 10, 20 and 30 o.c. .

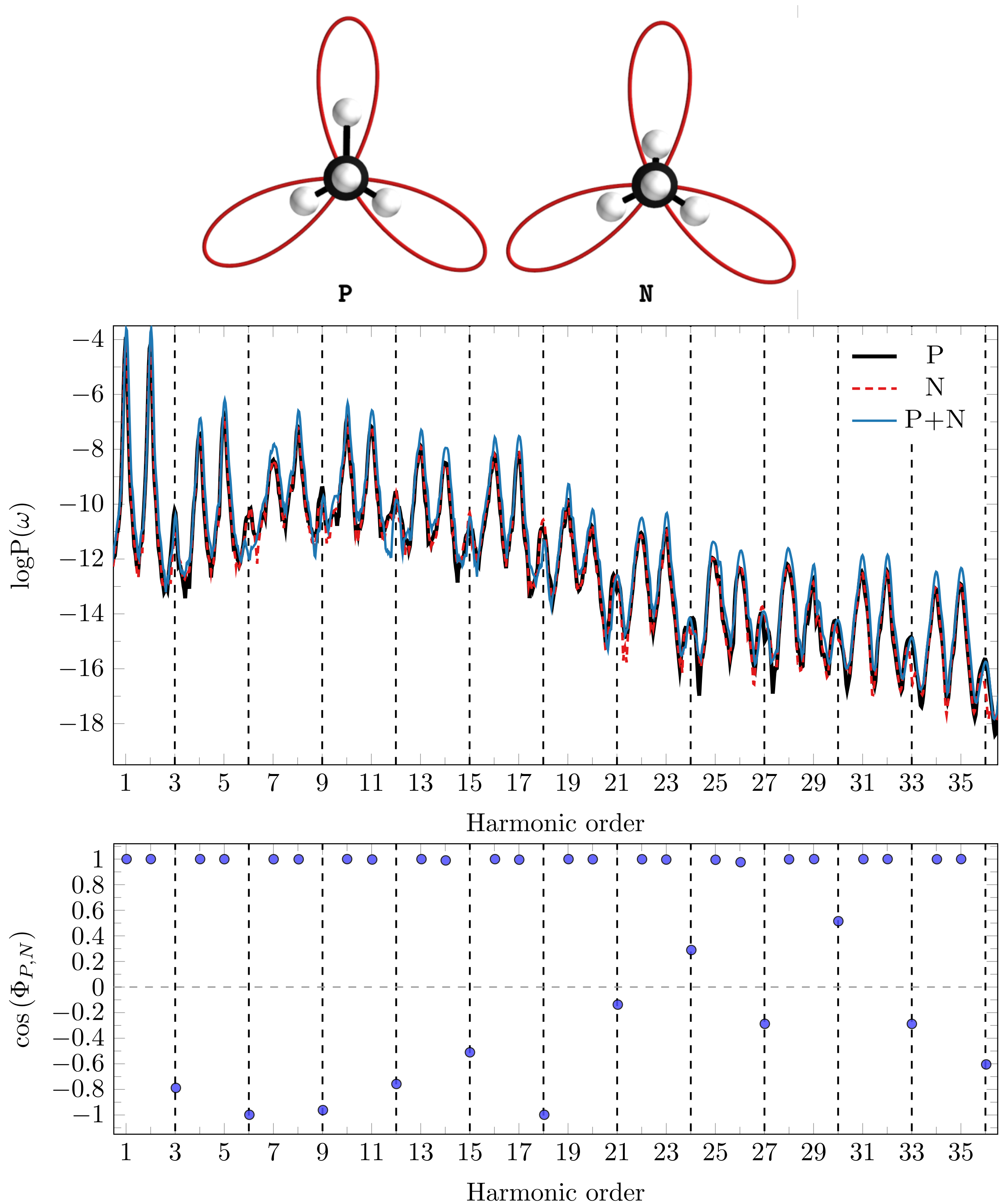

Figure S11: HHG spectra and interferences for structures P and N. Upper panel: Schematic representation of the complementary molecular configurations P and N. Middle panel: HHG spectra generated individually by configurations P and N, along with the coherent sum of their time-dependent dipoles. Bottom panel: phase difference between the dipole responses of the two configurations as a function of harmonic order.

References

(1) Coccia, E.; Luppi, E. Optimal-continuum and multicentered Gaussian basis sets for high-harmonic generation spectroscopy. *Theor. Chem. Acc.* **2016**, *135*, 43.

(2) Coccia, E.; Mussard, B.; Labeye, M.; Caillat, J.; Taïeb, R.; Toulouse, J.; Luppi, E. Gaussian continuum basis functions for calculating high-harmonic generation spectra. *Int. J. Quantum Chem.* **2016**, *116*, 1120.

(3) Marchetta, M.; Morassut, C.; Toulouse, J.; Coccia, E.; Luppi, E. Time-dependent ab initio molecular-orbital decomposition for high-harmonic generation spectroscopy. *J. Chem. Phys.* **2024**, *161*, 204111.

(4) Luppi, E.; Coccia, E. Role of Inner Molecular Orbitals in High-Harmonic Generation Spectra of Aligned Uracil. *J. Phys. Chem. A* **2023**, *127*, 7335.

(5) Morassut, C.; Ravindran, A.; Ciavardini, A.; Luppi, E.; De Ninno, G.; Coccia, E. High-Harmonic Generation Spectroscopy of Gas-Phase Bromoform. *J. Phys. Chem. A* **2024**, *128*, 2015.

(6) Marchetta, M.; Luppi, E.; Coccia, E. Dynamical symmetries and selection rules in high-harmonic generation spectroscopy of nonlinear molecules. *Phys. Chem. Chem. Phys.* **2025**, *27*, 12801.

(7) Hasović, E.; Odžak, S.; Becker, W.; Milošević, D. High-order harmonic generation in non-planar molecules driven by a bicircular field. *Mol. Phys.* **2017**, *115*, 1750.

(8) Stanton, J. F. A refined estimate of the bond length of methane. *Mol. Phys.* **1999**, *97*, 841.

(9) Shao, Y. et al. Advances in methods and algorithms in a modern quantum chemistry program package. *Phys. Chem. Chem. Phys.* **2006**, *8*, 3172.

(10) Coccia, E.; Luppi, E. Detecting the minimum in argon high-harmonic generation spectrum using Gaussian basis sets. *Theor. Chem. Acc.* **2019**, *138*, 96.

(11) Luppi, E.; Coccia, E. Probing the molecular frame of uracil and thymine with high-harmonic generation spectroscopy. *Phys. Chem. Chem. Phys.* **2021**, *23*, 3729.

(12) Coccia, E.; Luppi, E. Time-dependent ab initio approaches for high-harmonic generation spectroscopy. *J. Phys. Condens. Matter* **2021**, *34*, 073001.

(13) Woźniak, A. P.; Lesiuk, M.; Przybytek, M.; Efimov, D. K.; Prauzner-Bechcicki, J. S.; Mandrysz, M.; Ciappina, M.; Pisanty, E.; Zakrzewski, J.; Lewenstein, M.; Moszyński, R. A systematic construction of Gaussian basis sets for the description of laser field ionization and high-harmonic generation. *J. Chem. Phys.* **2021**, *154*, 094111.

(14) White, A. F.; Heide, C. J.; Saalfrank, P.; Head-Gordon, M.; Luppi, E. Computation of high-harmonic generation spectra of the hydrogen molecule using time-dependent configuration-interaction. *Mol. Phys.* **2016**, *114*, 947.

(15) Dunning, T. H. Gaussian basis sets for use in correlated molecular calculations. I. The atoms boron through neon and hydrogen. *J. Chem. Phys.* **1989**, *90*, 1007.

(16) Kaufmann, K.; Baumeister, W.; Jungen, M. Universal Gaussian basis sets for an optimum representation of Rydberg and continuum wavefunctions. *J. Phys. B* **1989**, *22*, 2223.

(17) Klinkusch, S.; Saalfrank, P.; Klamroth, T. Laser-induced electron dynamics including photoionization: A heuristic model within time-dependent configuration interaction theory. *J. Chem. Phys.* **2009**, *131*, 114304.

(18) Morassut, C.; Coccia, E.; Luppi, E. Quantitative performance analysis and comparison of optimal-continuum Gaussian basis sets for high-harmonic generation spectra. *J. Chem. Phys.* **2023**, *159*, 124108.
